# Ly6C^-^ Monocytes Regulate Parasite-Induced Liver Inflammation by Inducing the Differentiation of Pathogenic Ly6C^+^ Monocytes into Macrophages

**DOI:** 10.1371/journal.ppat.1004873

**Published:** 2015-05-28

**Authors:** Yannick Morias, Chloé Abels, Damya Laoui, Eva Van Overmeire, Martin Guilliams, Elio Schouppe, Frank Tacke, Carlie J. deVries, Patrick De Baetselier, Alain Beschin

**Affiliations:** 1 Myeloid Cell Immunology Laboratory, Vlaams Instituut voor Biotechnologie (VIB), Brussels, Belgium; 2 Cellular and Molecular Immunology Unit, Vrije Universiteit Brussel (VUB), Brussels, Belgium; 3 Inflammation Research Center, Vlaams Instituut voor Biotechnologie (VIB), Ghent, Belgium; 4 Laboratory of Immunoregulation and Mucosal Immunology, University Gent, Gent, Belgium; 5 Department of Medicine III, Rheinisch-Westfaelische Technische Hochschule (RWTH) University Hospital Aachen, Aachen, Germany; 6 Department of Medical Biochemistry, Academic Medical Center, University of Amsterdam, Amsterdam, The Netherlands; New York University, UNITED STATES

## Abstract

Monocytes consist of two well-defined subsets, the Ly6C^+^ and Ly6C^–^ monocytes. Both CD11b^+^ myeloid cells populations have been proposed to infiltrate tissues during inflammation. While infiltration of Ly6C^+^ monocytes is an established pathogenic factor during hepatic inflammation, the role of Ly6C^–^ monocytes remains elusive. Mice suffering experimental African trypanosome infection die from systemic inflammatory response syndrome (SIRS) that is initiated by phagocytosis of parasites by liver myeloid cells and culminates in apoptosis/necrosis of liver myeloid and parenchymal cells that reduces host survival. C57BL/6 mice are considered as trypanotolerant to *Trypanosoma congolense* infection. We have reported that in these animals, IL-10, produced among others by myeloid cells, limits the liver damage caused by pathogenic TNF-producing Ly6C^+^ monocytes, ensuring prolonged survival. Here, the heterogeneity and dynamics of liver myeloid cells in *T*. *congolense*-infected C57/BL6 mice was further dissected. Moreover, the contribution of Ly6C^–^ monocytes to trypanotolerance was investigated. By using FACS analysis and adoptive transfer experiments, we found that the accumulation of Ly6C^–^ monocytes and macrophages in the liver of infected mice coincided with a drop in the pool of Ly6C^+^ monocytes. Pathogenic TNF mainly originated from Ly6C^+^ monocytes while Ly6C^–^ monocytes and macrophages were major and equipotent sources of IL-10 within myeloid cells. Moreover, Nr4a1 (Nur77) transcription factor-dependent Ly6C^–^ monocytes exhibited IL-10-dependent and cell contact-dependent regulatory properties contributing to trypanotolerance by suppressing the production of TNF by Ly6C^+^ monocytes and by promoting the differentiation of the latter cells into macrophages. Thus, Ly6C^–^ monocytes can dampen liver damage caused by an extensive Ly6C^+^ monocyte-associated inflammatory immune response in *T*. *congolense* trypanotolerant animals. In a more general context, Ly6C^–^ or Ly6C^+^ monocyte targeting may represent a therapeutic approach in liver pathogenicity induced by chronic infection.

## Author Summary

The liver is not only a central organ for efficient metabolism of nutrients and for toxin clearance, but also for immune surveillance, including elimination of intravascular infections. However, excess of nutrients like fat or of toxins like alcohol and certain medications, as well as infections can trigger overactive immune responses which destroy the liver. Such chronic inflammations are major worldwide human health problem with often lethal consequences. Thus, understanding the particular function of various liver immune cells could provide original concepts to alleviate damages in this vital organ. Here, we dissected the heterogeneity, dynamics and function of the myeloid/monocytic cell compartment in the liver of mice infected with *Trypanosoma congolense* parasite. We established that infiltration of Ly6C^+^ monocyte subset initiated liver injury in infected mice. More importantly, we revealed that another myeloid cell subset for which the role in liver injury remained elusive, the Ly6C^-^ monocyte subset, exerted hepatoprotective function in infected mice by secreting the anti-inflammatory cytokine IL-10 and by inducing, through cell-contact, the differentiation of pathogenic Ly6C^+^ monocytes into macrophages expressing genes coding for anti-inflammatory molecules. Thus, augmenting Ly6C^-^ monocyte accumulation or functionality may represent a useful intervention strategy complementing anti-infective medication in conditions of liver injury due to chronic infections.

## Introduction

Hosts can develop two different strategies to control pathogen infections, resistance and tolerance. During resistance, the host reduces the pathogen burden by activating and recruiting immune cells to the site of infection that mount a pro-inflammatory immune response. Tolerance refers to the action whereby the host repairs the tissue damage, i.e the pathogenicity, caused by the inflammatory immune cells that mediate the resistance [1, 2]. African trypanosomes are extracellular protozoan parasites causing sleeping sickness in humans and Nagana disease in cattle in sub-Saharan Africa. In experimental *Trypanosoma congolense* infection, C57BL/6 mice are considered as "trypanotolerant", being resistant and tolerant to the disease. The resistance of these animals results from their capacity to develop IFN-γ and MyD88-dependent CD11b^+^ myeloid cells, i.e. M1-type myeloid cells, including CCR2-dependent Ly6C^+^ monocytes and macrophages that secrete trypanotoxic molecules like TNF and NO and exert phagocytic activity to control the parasitemia [3–9]. This control of parasite growth occurs mainly in the liver [4, 10]. However, the M1-activated Ly6C^+^ monocyte subpopulation negatively affects the tolerance to *T*. *congolense* infection. Indeed, *T*. *congolense*-infected mice die from a systemic inflammatory response syndrome that is initiated by the engulfment of parasite components by liver myeloid cells, and culminates in apoptosis of liver myeloid cells and necrosis of liver parenchymal cells that reduce the host survival [3, 4]. In this respect, Ly6C^+^ monocytes have been shown to trigger liver cell apoptosis/necrosis, resulting in organ failure and early death by perpetuating a TNF-mediated pro-inflammatory immune response [7, 11]. On the other hand, IL-10 does not influence the resistance but is an essential contributor to the tolerance to *T*. *congolense* infection. This cytokine has been shown to down-regulate the Ly6C^+^ monocyte-induced pathogenicity and to induce regulatory, M2-type myeloid cells expressing a number of genes that could contribute to tissue healing, including maintenance of liver homeostasis. Both regulatory T cells and CD11b^+^ myeloid cells have been identified as sources of IL-10 during *T*. *congolense* infection in trypanotolerant animals [7, 10, 11]. Yet, within the heterogeneous CD11b^+^ myeloid cell population, the subset responsible for the IL-10 mediated anti-inflammatory immune response, thus for trypanotolerance, remained to be identified. In this study, we reveal the mobilization of IL-10-expressing Ly6C^-^ monocytes and macrophages after the control of the first peak of parasitemia when a M2-type regulatory immune response arises in the liver of *T*. *congolense*—infected mice. In particular, Ly6C^-^ monocytes exerted regulatory functions through the suppression of TNF production by Ly6C^+^ monocytes and by promoting the differentiation of the latter monocytes into macrophages. Hereby, Ly6C^-^ monocytes contributed to the trypanotolerance by dampening liver damage caused by *T*. *congolense*—induced pro-inflammatory immune response. Thus, while the liver protective function of IL-10-producing regulatory T cells is amply documented [12–14], we have now established a similar function for Ly6C^-^ monocytes.

## Results

### Trypanosome infection induces the sequential mobilization of distinct myeloid cell subsets in the liver

**Fig 1:**
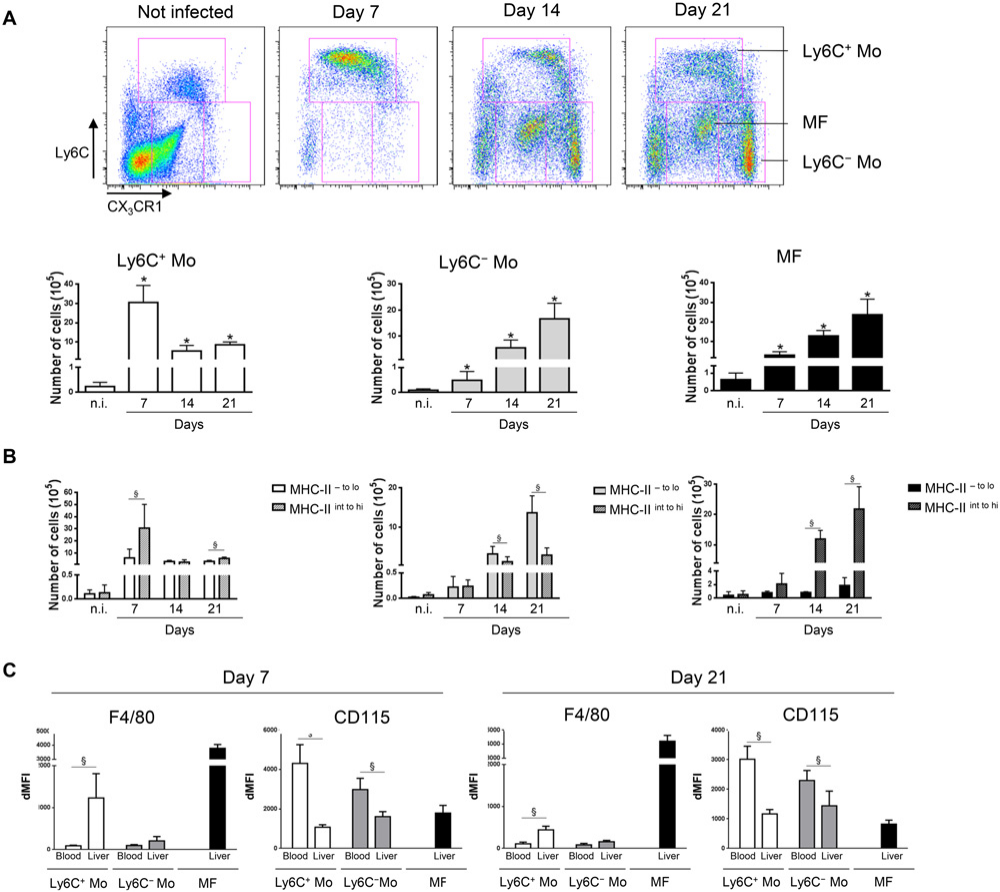
Distinct liver myeloid cell subsets are sequentially mobilized in *T*. *congolense*-infected mice. Ly6C^+^ monocytes (Ly6C^+^ Mo), Ly6C^-^ monocytes (Ly6C^-^ Mo) and macrophages (MF) were gated based on Ly6C and CX_3_CR1/GFP expression as illustrated in S1A Fig in S1 Text in non-infected (n.i) and infected CX_3_CR1-GFP^+/-^ mice at day 7, 14 and 21 pi. Numbers of **(A)** the 3 myeloid cell subsets and **(B)** of the MHC-II^- to lo^ and MHC-II^int to hi^ fraction of Ly6C^+^ monocytes, Ly6C^-^ monocytes and macrophages within liver non-parenchymal cell population were determined. Data are shown as mean + SD of 3 individual mice from one representative out of four independent experiments. * p< 0.05 compared to non-infected mice; § p<0.05 comparing populations linked by horizontal bar. **(C)** F4/80 or CD115 expression on Ly6C^+^ monocytes, Ly6C^-^ monocytes and macrophages from the blood or the liver at day 7 and 14 pi determined as mean fluorescence intensity difference (dMFI) between anti-F/80 or anti-CD115 and isotype control antibodies.

The phenotype of intrahepatic CD11b^+^ myeloid cells during parasite-induced liver inflammation was investigated in CX_3_CR1-GFP^+/-^ C57BL/6 mice infected with *T*. *congolense* at day 7, 14 and 21 post infection (pi). Based on FACS analysis (Fig 1A, S1 Fig in S1 Text), three main cell subsets were identified in the liver of infected mice: Ly6C^+^ 'inflammatory' monocytes (CX_3_CR1^int^ CD11b^hi^ CD115^hi^ MHC-II^- to int^ CD62L^hi^ F4/80^int^ Mertk^-^ CD64^lo^ CD11c^-^ Mar-1^-^), Ly6C^-^ 'patrolling' monocytes (CX_3_CR1^hi^ CD11b^hi^ CD115^hi^ MHC-II^- to lo^ CD11a^hi^ F4/80^lo^ Mertk^-^ CD64^-^ CD11c^int^ Mar-1^-^) and macrophages (Ly6C^-^ CD11b^int^ CX3CR1^int^ F4/80^hi^ Mertk^+^ MHC-II^hi^ CD115^lo^ CD64^hi^ CD11c^-^ Mar-1^-^) [15–19]. When addressing the dynamics of these three distinct liver myeloid cell subsets, Ly6C^+^ monocytes were found to be recruited predominantly at day 7 pi (Fig 1A) when a M1-type inflammatory immune response is mounted to control the first peak of parasitemia [4, 6], while the Ly6C^-^ monocytes and the macrophages accumulated in the late stage of infection at day 21 pi (Fig 1A), when a M2-type/regulatory immune response develops and controls the liver damage caused by the M1-type response [7, 11]. Moreover, the Ly6C^+^ monocytes and the macrophages mobilized in the liver of infected mice were prominently MHC-II^- to int^ and MHC-II^int to hi^, respectively, while the MHC-II^- to lo^ fraction of the Ly6C^-^ monocytes accumulated (Fig 1B, S2 Fig in S1 Text). As compared to blood monocytes, liver Ly6C^+^ monocytes showed an increased F4/80 and MHC-II expression and a decreased CD115 expression (Fig 1C, S2 Fig in S1 Text), suggesting their maturation upon entering the liver of *T*. *congolense*-infected mice. Blood Ly6C^-^ monocytes only exhibited decreased CD115 expression upon entry in the liver (Fig 1C, S2 Fig in S1 Text).

### Ly6C^+^ monocytes differentiate into macrophages in the liver of *T*. *congolense*-infected mice

**Fig 2:**
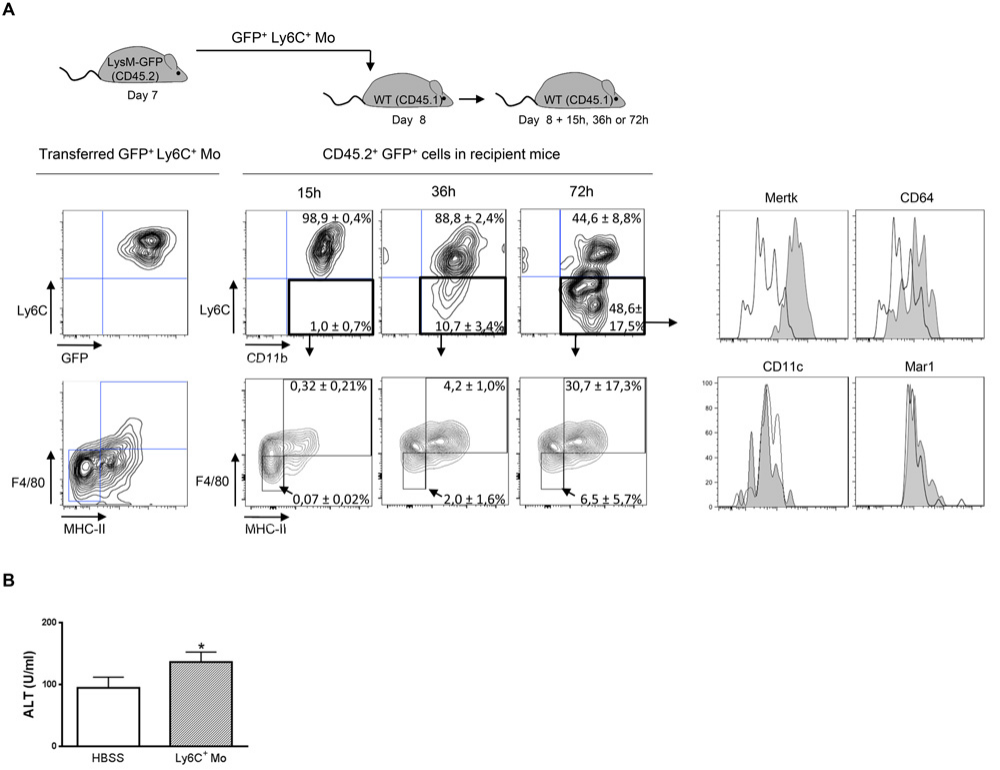
Ly6C^+^ monocytes differentiate into macrophages in *T*. *congolense*-infected mice. **(A)** Liver GFP^+^ Ly6C^+^ monocytes (Ly6C^+^ Mo) purified from CD45.2 LysM-GFP mice at day 7 pi were transferred in CD45.1 WT mice at d8 pi. After 15, 36 and 72 h, liver CD45.2^+^ GFP^+^ cells from recipient mice were analysed for CD11b and Ly6C expression. MHC-II, F4/80, Mertk, CD64, CD11c and Mar1 expression was then investigated in Ly6C^-^ CD11b^+^ cells. FACS profiles are representative of 1 out of 9 mice tested in three independent experiments. Percentages of cells in indicated gates are as shown as mean ± SD of 3 individual mice of one representative out of three independent experiments. **(B)** ALT levels in mice treated with Ly6C^+^ monocytes or with HBSS as control. Data are shown as mean + SD of 3 individual mice of one representative out of three independent experiments. * p<0.05 compared to control mice.

In view of the sequential accumulation of myeloid cell subsets in the liver of infected mice (Fig 1), the turnover of Ly6C^+^ and Ly6C^-^ monocytes and their relationship with the macrophage pool was investigated. When BrdU was continuously administered in infected mice, blood Ly6C^+^ monocytes became more rapidly BrdU^+^ than liver Ly6C^+^ monocytes concomitant with the acquisition of Ki67 expression (S3 Fig in S1 Text), indicating a rapid turnover and/or proliferation rate of the circulating Ly6C^+^ monocytes. In comparison, liver macrophages and even more blood and liver Ly6C^-^ monocytes showed a longer lag-phase in BrdU incorporation and a KI67^lo^ and Ki67^-^ profile, respectively, pointing to a lower turnover and/or proliferation rate of the 2 latter cell populations (S3 Fig in S1 Text). These data suggest that BrdU^+^ Ly6C^+^ monocytes can differentiate into a macrophage and/or a Ly6C^-^ monocyte population in the liver of infected mice. Accordingly, when Ly6C^+^ monocytes isolated from the liver of *T*. *congolense*—infected CD45.2 LysM-GFP mice (in which myeloid cells that express LysM are labeled with GFP; [20]) were transferred in infected CD45.1 wild-type (WT) mice, they mainly acquired within 72 h, a CD11b^int^ Ly6C^-^ MHC-II^+^ F4/80^hi^ CD64^lo^ Mertk^hi^ but CD11c^-^ and Mar-1^-^ profile (Fig 2A) representing macrophages (S1 Fig in S1 Text; [16, 18, 19]). In contrast, Ly6C^-^ monocytes from *T*. *congolense*—infected CX_3_CR1-GFP^+/-^ CD45.2 mice transferred in CD45.1 mice maintained within 96 h post-transfer a CX_3_CR1^hi^ Ly6C^-^ profile and did not acquire F4/80 and MHC-II expression (S4 Fig in S1 Text). A minor fraction of the transferred GFP^+^ Ly6C^+^ monocytes acquired a Ly6C^-^ F4/80^lo^ MHC-II^-^ phenotype (Fig 2A), similar to the Ly6C^-^ monocyte phenotype (S1 Fig in S1 Text). These data and the longest lag-phase in BrDU incorporation for Ly6C^-^ monocytes observed in S3 Fig in S1 Text, suggested that Ly6C^+^ monocytes could differentiate into Ly6C^-^ monocytes during *T*. *congolense*-induced inflammation. Thus, we investigated whether the reduction of circulating and liver Ly6C^+^ monocytes occurring in CCR2^-/-^ mice infected with African trypanosome [21] affected the presence of the other liver myeloid cell subsets. The absence of GFP reporter gene in the CCR2^-/-^ mice at our disposition required the setting of an alternative gating strategy to distinguish the Ly6C^+^ monocyte, the Ly6C^-^ monocyte and the macrophage cell subsets (S5A Fig in S1 Text). This strategy was (i) based on the differential expression of CD115, Ly6C, MHC-II and F4/80 molecules by these three cell populations reported in S1 Fig in S1 Text, and (ii) validated using CX_3_CR1-GFP^+/-^ mice (S5B Fig in S1 Text). At day 7 and 21 pi, *T*. *congolense*-infected CCR2^-/-^ mice exhibited not only a lower amount of Ly6C^+^ monocytes, but also of Ly6C^-^ monocytes in the blood and the liver (S5C Fig in S1 Text), confirming that some Ly6C^+^ monocytes can differentiate into Ly6C^-^ monocytes in infected mice. The reduced amount of monocytes in infected CCR2^-/-^ mice coincided with a trend towards lower amount of liver macrophages (S5CFig in S1 Text). Together, these data indicate that Ly6C^+^ monocytes differentiate mainly into macrophages but also into Ly6C^-^ monocytes in the liver of *T*. *congolense*-infected mice. In contrast, Ly6C^-^ monocytes did not acquire macrophage differentiation markers.

### Liver myeloid cell subsets from trypanosome-infected mice exhibit different activation status

**Fig 3:**
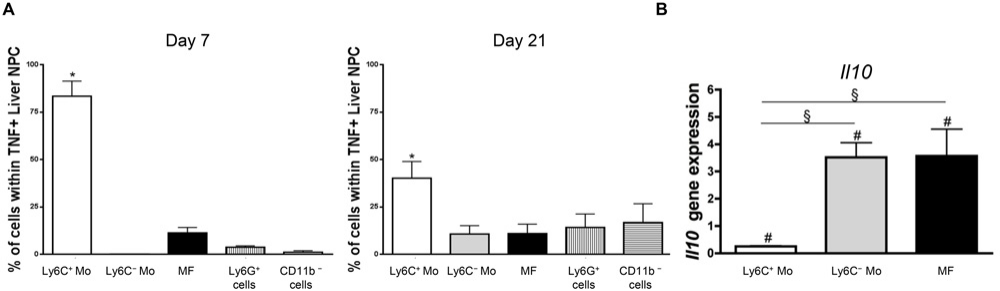
Ly6C^+^ monocytes are the main producers of TNF while Ly6C^-^ monocytes and macrophages are the major source of IL-10 in *T*. *congolense*-infected mice. **(A)** Intracellular TNF^+^ cells were gated in liver non-parenchymal cells from CX_3_CR1-GFP^+/-^ mice at day 7 and 21 pi. The percentage of Ly6C^+^ monocytes (Ly6C^+^ Mo), Ly6C^-^ monocytes (Ly6C^-^ Mo), macrophages (MF) gated as in Fig 1A, and of CD11b^+^ Ly6G^+^ neutrophils and CD11b^-^ cell was determined within TNF^+^ cells. Data are shown as mean + SD of 3 individual mice from one representative out of three independent experiments. * p<0.05 compared to other populations **(B)** *Il10* gene expression in liver Ly6C^+^ monocytes, Ly6C^-^ monocytes and macrophages purified from CX_3_CR1-GFP^+/-^ mice at day 21 pi was normalized against *S12* gene expression and expressed relatively to gene expression in non-fractionated CD11b^+^ liver cells. Data are shown as mean + SD of 3 individual mice from one representative out of three independent experiments. # p<0.05 compared to non-fractionated CD11b^+^ myeloid cells; § p<0.05 comparing populations linked by horizontal bar.

The transfer of Ly6C^+^ monocytes at day 6 pi increased liver injury reflected by increased alanine aminotransferase (ALT) serum levels in *T*. *congolense*-infected mice (Fig 2B), confirming the pathogenic nature of these cells [21]. These cells were the major source of TNF in the liver of infected mice (Fig 3A). Our previous research established that liver CD11b^+^ Ly6G^-^ myeloid cells contributed to IL-10 production and expressed genes associated with a M2-type activation, hereby limiting the production of hepatotoxic TNF by Ly6C^+^ monocytes [7, 10, 21]. In view of the expansion of both the Ly6C^-^ monocyte and the macrophage populations within liver CD11b^+^ myeloid cells at this stage of *T*. *congolense* infection (Fig 1A), their relative contribution to IL-10 production and M2-type activation was investigated at day 21 pi. As compared to non-fractionated CD11b^+^ myeloid cells, the *Il10* gene expression was similarly increased in Ly6C^-^ monocytes and macrophages, while down-regulated in Ly6C^+^ monocytes (Fig 3B). Moreover, investigating the expression of genes that are induced by IL-10 in M2-type non-fractionated CD11b^+^ cells during African trypanosome infection [7, 10, 22], showed that *Folr2*, *Ctss*, *Mgl2* and *Sepp1* expression was upregulated in macrophages but not in the other myeloid cell subsets from *T*. *congolense*—infected mice; *Ngfb* and *Arg1* expression was induced to similar levels in the three myeloid cell subpopulations; and *F13a1* expression was induced at higher level in Ly6C^+^ monocytes than in the other myeloid cell subpopulations (S6A Fig in S1 Text). Concerning M1-type genes triggered in non-fractionated CD11b^+^ myeloid from *T*. *congolense*—infected mice [7, 10], *Tnf* and to lower extent *Nos2* expression was upregulated mainly in Ly6C^+^ monocytes, and *Ccl2*, *Cxcl9* and *Cxcl10* expression was induced in macrophages. With the exception of *Ccl2*, none of the tested genes showed induced expression in Ly6C^-^ monocytes compared to the non-fractionated CD11b^+^ myeloid cell population (S6B Fig in S1 Text). These data suggest that Ly6C^-^ monocytes and macrophages were the main IL-10 producers within liver CD11b^+^ myeloid cell subsets in the late stage of *T*. *congolense* infection. Moreover, the three myeloid cell subsets showed differential activation status, Ly6C^+^ monocytes expressing mainly M1-type genes, Ly6C^-^ monocytes expressing mainly IL-10 and macrophages expressing a mix of M1/M2-type genes.

### Ly6C^-^ monocytes limit the production of TNF by Ly6C^+^ monocytes during trypanosome-induced liver injury

The expansion of IL-10-producing Ly6C^-^ monocytes and the decline of pathogenic TNF-producing Ly6C^+^ monocytes in late stage *T*. *congolense*—infected mice prompted us to investigate whether the former monocytes exerted anti-inflammatory/hepatoprotective activity. We focused on the role of the MHC-II^- to lo^ Ly6C^-^ monocyte fraction since this population was found to accumulate in the course of infection (Fig 1B; S2 Fig in S1 Text).

**Fig 4:**
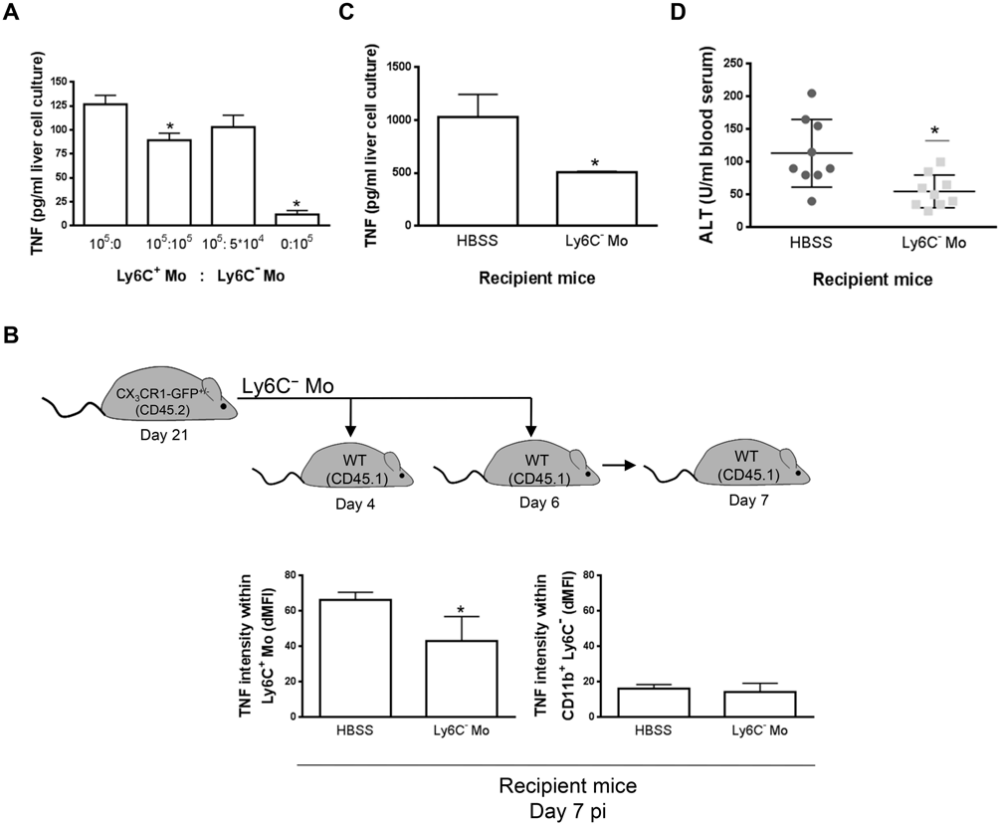
Ly6C^-^ monocytes limit TNF production by Ly6C^+^ monocytes in *T*. *congolense*-infected mice. **(A)** Liver Ly6C^+^ monocytes (Ly6C^+^ Mo) and MHC-II^- to lo^ Ly6C^-^ monocytes (Ly6C^-^ Mo) purified from CX_3_CR1-GFP^+/-^ mice at day 7 pi and 21 pi, respectively, were cultured at indicated ratio. TNF concentration in cell supernatants was measured. Data are shown as mean + SD of 1 representative out of five independent experiments. * p<0.05 compared to Ly6C^+^ monocytes cultured alone. **(B-D)** Liver MHC-II^- to lo^ Ly6C^-^ monocytes purified from CD45.2 CX_3_CR1-GFP^+/-^ mice at day 21 pi were transferred in CD45.1 WT mice at day 4 and day 6 pi. Control mice received HBSS. Different parameters were evaluated in recipient mice at d7 pi: **(B)** Spontaneous TNF levels in liver Ly6C^+^ monocytes **(left panel)** and CD11b^+^ Ly6C^-^ cells **(right panel)** from control or Ly6C^-^ monocyte-treated mice determined as mean fluorescence intensity difference (dMFI) between anti-TNF and isotype control antibodies. **(C)** Spontaneous TNF concentration in supernatants of liver non-parenchymal cell cultures and **(D)** serum alanine aminotransferase (ALT) levels. Data are shown as mean + SD of 3 individual mice of one representative out of three independent experiments. * p<0.05 compared to control mice.

**Fig 5:**
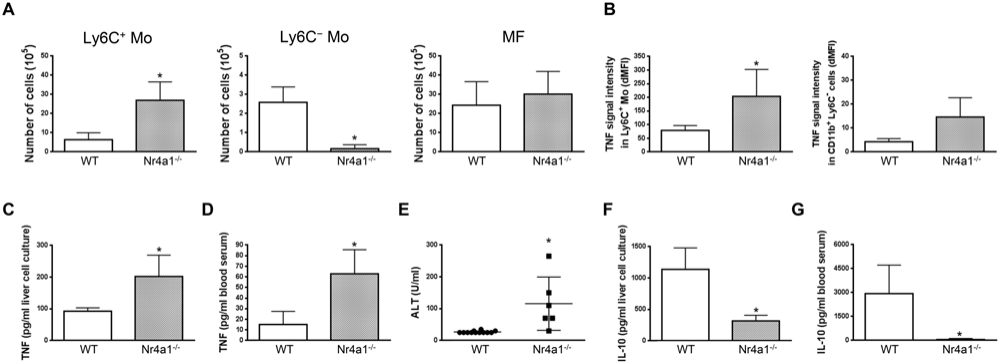
Depletion of Ly6C^-^ monocytes in Nr4a1^-/-^ mice increases TNF production and liver damage in *T*. *congolense*—infected mice. Different parameters were evaluated in WT and Nr4a1^-/-^ mice at day 21 pi. Liver Ly6C^+^ monocytes (Ly6C^+^ Mo), Ly6C^-^ monocytes (Ly6C^-^ Mo) and macrophages (MF) were gated as in S5A Fig in S1 Text. **(A)** Numbers of Ly6C^+^ monocytes, Ly6C^-^ monocytes and macrophages within liver non-parenchymal cell population. **(B)** Spontaneous TNF levels in Ly6C^+^ monocytes and CD11b^+^ Ly6C^-^ cells determined as mean fluorescence intensity difference (dMFI) between anti-TNF and isotype control antibodies. **(C,D)** TNF and **(F,G)** IL-10 concentration in **(C,F)** supernatants of liver non-parenchymal cell cultures or **(D,G)** in blood serum. **(E)** ALT levels in blood serum. Data are shown as mean + SD of 3 individual mice of one representative out of five independent experiments. *p<0.05 compared to WT mice.

In *in vitro* co-cultures, Ly6C^-^ monocytes purified from the liver at day 21 pi were found to reduce the production of TNF by liver Ly6C^+^ monocytes isolated at day 7 pi (Fig 4A), when their production of TNF was maximal [7, 10, 11]. To validate these data *in vivo*, Ly6C^-^ monocytes were sorted from *T*. *congolense*-infected CD45.2 CX_3_CR1-GFP^+/-^ mice at day 21 pi and transferred in CD45.1 WT mice at day 4 and 6 pi. At day 7 pi, Ly6C^+^ monocytes from recipient mice expressed a higher intracellular level of TNF than the CD11b^+^ Ly6C^-^ cell fraction (Fig 4B) that includes macrophages and Ly6C^-^ monocytes (S1 Fig in S1 Text). The two latter cell populations are thus marginal contributors to TNF production as compared to Ly6C^+^ monocytes in infected mice (in agreement with Fig 3A). More importantly, the intracellular production of TNF decreased in the Ly6C^+^ monocyte but not in the CD11b^+^ Ly6C^-^ cell fraction in recipient mice treated with Ly6C^-^ monocytes as compared to control-treated mice (Fig 4B). The decrease in TNF production in Ly6C^+^ monocytes from recipient mice treated with Ly6C^-^ monocytes coincided with a reduced production of TNF in non-parenchymal cell culture supernatants and reduced liver damage (Fig 4C and 4D). These data suggest that Ly6C^-^ monocytes restrained TNF production by Ly6C^+^ monocytes and hereby exerted hepatoprotective function in *T*. *congolense*—infected mice. To support these data obtained by augmenting the Ly6C^-^ population in infected animals through adoptive transfer experiments, we adopted a reverse strategy, using Nr4a1^-/-^ mice in which Ly6C^-^ monocytes are almost absent from the circulation due to apoptosis in the bone marrow [23]. Non-parenchymal liver cells from Nr4a1^-/-^ and WT mice cultured ex-vivo secreted similar amount of TNF. Moreover, in both mouse strains, the percentage of TNF producing Ly6C^+^ monocytes and macrophages as well as their respective TNF production were comparable (S7 Fig in S1 Text), confirming that the Nr4a1 transcription factor did not affect the capacity of Ly6C^+^ monocytes and macrophages to secrete TNF [24]. In *T*. *congolense*—infected Nr4a1^-/-^ mice, the reduction of the liver Ly6C^-^ monocyte population coincided with the increased accumulation of Ly6C^+^ monocytes (Fig 5A). These cells produced more TNF (Fig 5B), resulting in increased production of TNF in non-parenchymal cell culture supernatants as well as in the blood (Fig 5C and 5D), coupled with increased ALT serum levels (Fig 5E). Moreover, although the percentage of liver macrophages decreased in Nr4a1^-/-^ mice (S8 Fig in S1 Text), no difference in their absolute number was observed (Fig 5A) due to the higher amount of liver non-parenchymal isolated from infected Nr4a1^-/-^ mice. The TNF production by CD11b^+^ Ly6C^-^ cells that include mainly macrophages marginally increased (Fig 5B). Furthermore, the lack of Ly6C^-^ monocytes and the decrease in percentage of the macrophage population in the liver of infected Nr4a1^-/-^ mice associated with reduced IL-10 levels in cell culture supernatants and in blood (Fig 5F and 5G), confirming these two cell types as potential sources of IL-10 in *T*. *congolense*—infected mice (Fig 3B).

### IL-10 contributes to the Ly6C^-^ monocyte-regulatory activity during trypanosome infection

Ly6C^-^ monocytes could contribute to the production of IL-10 in *T*. *congolense*—infected mice (Fig 3; Fig 5F and 5G) and limit the production of TNF by Ly6C^+^ monocytes (Fig 4A–4C; Fig 5B–5D). Therefore, we investigated whether the limitation of TNF production by Ly6C^-^ monocytes was mediated by IL-10. Two types of experiments were performed.

**Fig 6:**
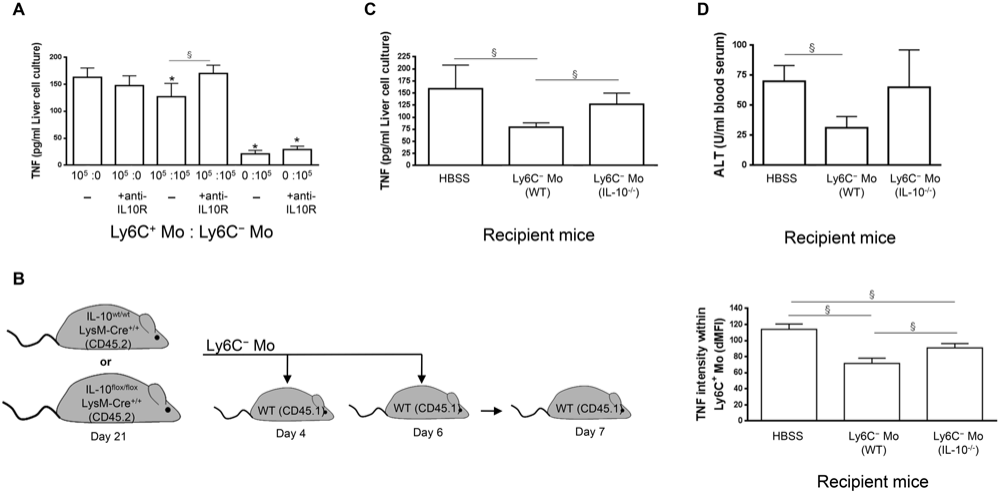
Ly6C^-^ monocytes through IL-10 production limit TNF production by Ly6C^+^ monocytes in *T*. *congolense*-infected mice. **(A)** Liver Ly6C^+^ monocytes (Ly6C^+^ Mo) and MHC-II^- to lo^ Ly6C^-^ monocytes (Ly6C^-^ Mo) purified from CX_3_CR1-GFP^+/-^ mice at day 7 pi and 21 pi, respectively, were cultured alone or together at indicated ratio in presence of control (-) or neutralising anti-IL-10R antibody. TNF concentration in cell supernatants was measured. Data are shown as mean + SD of 1 out of five independent experiments. * p<0.05 compared to Ly6C^+^ monocytes cultured alone, § p<0.05 comparing populations linked by horizontal bar. **(B-D)** Liver MHC-II^- to lo^ Ly6C^-^ monocytes gated as described in Fig 5A in S1 Text purified from IL-10^wt/wt^ LysM-cre^+/+^ or IL-10^flox/flox^ LysM-cre^+/+^ mice at day 21 pi were transferred in CD45.1 WT mice at day 4 and day 6 pi. Control mice received HBSS. Different parameters were evaluated in recipient mice at day 7 pi: **(B)** TNF levels in liver Ly6C^+^ monocytes determined as mean fluorescence intensity difference (dMFI) between anti-TNF and isotype control antibodies, **(C)** Spontaneous TNF concentration in liver non-parenchymal cell culture supernatants, and **(D)** ALT levels in blood serum. Data are shown as mean + SD of 3 individual mice of one representative out of three independent experiments. § p<0.05 comparing populations linked by horizontal bar.

First, TNF production was monitored in co-cultures containing liver Ly6C^-^ and Ly6C^+^ monocytes sorted from mice at day 21 and day 7 pi, respectively, in the presence of neutralizing anti-IL-10R antibody (Fig 6A). Blocking the IL-10R signaling ablated the limitation of TNF production observed when Ly6C^+^ monocytes were cultured with Ly6C^-^ monocytes. In contrast, IL-10R signaling neutralization had no effect on the production of TNF when Ly6C^+^ monocytes were cultured alone.

Second, liver Ly6C^-^ monocytes were isolated at day 21 pi from CD45.2 *T*. *congolense*—infected IL-10^flox/flox^ LysM-cre^+/+^ mice, which lack IL-10 production specifically in LysM-expressing myeloid cells and hereby exhibit increased TNF levels and increased liver damage [7, 25]. These cells were adoptively transferred in CD45.1 WT mice at day 4 and 6 pi and the intracellular production of TNF by liver Ly6C^+^ monocytes was investigated at day 7 pi. As shown in Fig 6B, TNF production was impaired to a greater extent in recipient mice treated with Ly6C^-^ monocytes from control IL-10^wt/wt^ LysM-cre^+/+^ mice than in recipient mice treated with Ly6C^-^ monocytes from IL-10^flox/flox^ LysM-cre^+/+^ mice. Moreover, the secretion of TNF was reduced in cell culture supernatants of recipient mice treated with Ly6C^-^ monocytes from control IL-10^wt/wt^ LysM-cre^+/+^ mice but not from IL-10^flox/flox^ LysM-cre^+/+^ mice (Fig 6C). Finally, ALT levels were reduced in recipient mice treated with Ly6C^-^ monocytes from control IL-10^wt/wt^ LysM-cre^+/+^ mice but not from IL-10^flox/flox^ LysM-cre^+/+^ mice (Fig 6D). Together these data suggest that Ly6C^-^ monocytes could hamper TNF production by Ly6C^+^ monocytes partly via their IL-10 production and hereby reduce liver injury in *T*. *congolense*—infected mice.

### Ly6C^-^ monocytes promote the differentiation of Ly6C^+^ monocytes into macrophages during trypanosome-induced liver inflammation

**Fig 7:**
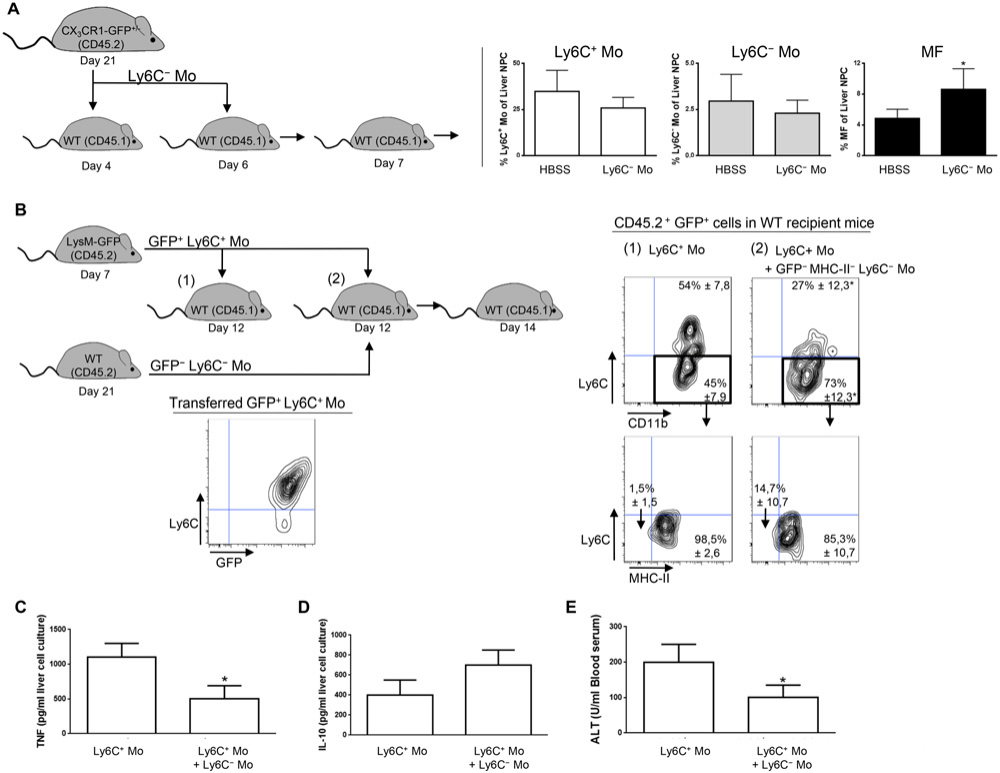
Ly6C^-^ monocytes induce differentiation of Ly6C^+^ monocytes into macrophages in *T*. *congolense*-infected WT mice. **(A)** Liver MHC-II^- to lo^ Ly6C^-^ monocytes (Ly6C^-^ Mo) purified from CX_3_CR1-GFP^+/-^ mice at day 21 pi were transferred in CD45.1 WT mice at day 4 and day 6 pi. Control mice received HBSS. At day 7 pi, percentages of Ly6C^+^ monocytes (Ly6C^+^ Mo), Ly6C^-^ monocytes (Ly6C^-^ Mo) and macrophages (MF) within liver non-parenchymal cells were determined in recipient mice. Data are shown as mean + SD of 3 individual mice of one representative out of three independent experiments. *p<0.05 compared to control mice. **(B)** GFP^+^ Ly6C^+^ monocytes purified from CD45.2 LysM-GFP mice at day 7 pi were transferred in CD45.1 WT mice at day 12 pi with or without MHC-II^-to lo^ Ly6C^-^ monocytes gated as described in S5A Fig in S1 Text and purified from CD45.2 WT mice at day 21 pi. After 48 h, liver CD45.2^+^ GFP^+^ cells in recipient mice were analyzed for CD11b and Ly6C expression. MHC-II expression was then investigated in Ly6C^-^ CD11b^+^ cells. FACS profiles are representative of 1 out of 9 mice tested in three independent experiments. Percentages of cells in indicated gates are shown as mean ± SD of 3 individual mice of one representative out of three independent experiments. **(C)** TNF and **(D)** IL-10 concentration in supernatants of liver non-parenchymal cell cultures from recipient mice. **(E)** ALT levels in blood serum from recipient mice. Data are shown as mean + SD of 3 individual mice of one representative out of three independent experiments. *p<0.05 compared to recipient mice receiving only Ly6C^+^ monocytes.

**Fig 8:**
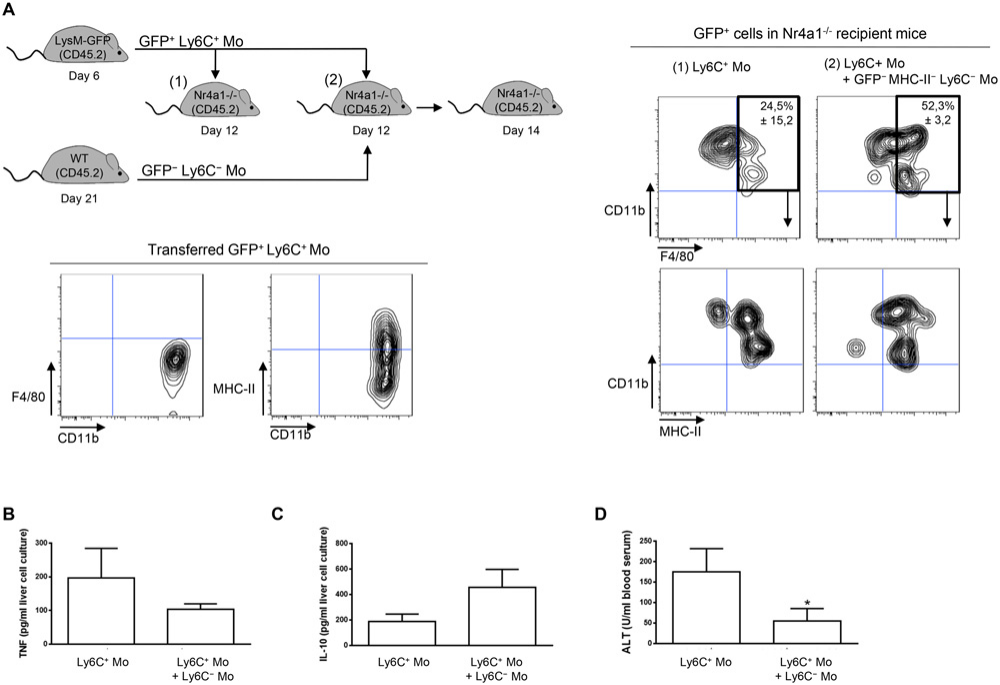
Ly6C^-^ monocytes induce differentiation of Ly6C^+^ monocytes into macrophages in *T*. *congolense*-infected Nr4a1^-/-^ mice that lack endogenous Ly6C^-^ monocytes. **(A)** GFP^+^ Ly6C^+^ monocytes (Ly6C^+^ Mo) purified from CD45.2 LysM-GFP mice at day 7 pi were transferred in CD45.2 Nr4a1^-/-^ mice at day 12 pi with or without MHC-II^-to lo^ Ly6C^-^ monocytes (Ly6C^-^ Mo) gated as described in Fig 5A in S1 Text and purified from CD45.2 WT mice at day 21 pi. After 48 h, liver GFP^+^ cells in recipient mice were analyzed for CD11b and F4/80 expression. MHC-II expression was then investigated in CD11b^+^ F4/80^+^ cells. FACS profiles are representative of 1 out of 9 mice tested in three independent experiments. Percentages of cells in indicated gates are shown as mean ± SD of 3 individual mice of one representative out of three independent experiments. **(B)** TNF and **(C)** IL-10 concentration in supernatants of liver non-parenchymal cell cultures from recipient mice. **(D)** ALT levels in blood serum from recipient mice. Data are shown as mean + SD of 3 individual mice of one representative out of three independent experiments. *p<0.05 compared to recipient mice receiving only Ly6C^+^ monocytes.

Transfer experiment of Ly6C^+^ monocytes in recipient mice suggested that a fraction of the liver macrophages in *T*. *congolense*—infected mice originate from Ly6C^+^ monocytes (Fig 2A). Moreover, the liver macrophage population reduced while the Ly6C^+^ cell population increased in infected Nr4a1^-/-^ mice lacking Ly6C^-^ monocytes (S8 Fig in S1 Text). In contrast, in infected mice treated with Ly6C^-^ monocytes, the Ly6C^-^ CX_3_CR1^int^ macrophage population increased (Fig 7A). Therefore, we investigated whether Ly6C^-^ monocytes shaped by *T*. *congolense* infection could favor the differentiation of Ly6C^+^ monocytes into macrophages. We used Ly6C^-^ monocytes from day 21 pi because their clear accumulation at that stage of infection coincided with increased macrophage accumulation (Fig 1A) and with a shift from a pathogenic to anti-pathogenic immune response required for trypanotolerance [7, 10, 11]. GFP^+^ Ly6C^+^ monocytes isolated from LysM-GFP mice at day 7 pi were adoptively transferred, with or without Ly6C^-^ monocytes isolated from CD45.2 mice at day 21 pi, in recipient CD45.1 WT mice at day 12 pi. Liver CD45.2^+^ GFP^+^ cells were characterized 48 h later (Fig 7B). In mice that were not treated with Ly6C^-^ monocytes, 45 ± 4.6% of the GFP^+^ monocytes differentiated into Ly6C^-^ CD11b^int^ MHC-II^+^ cells (Fig 7B) that represented macrophages (S1 Fig in S1 Text). These percentages increased to 73 ± 8.7% in mice treated with Ly6C^-^ monocytes (Fig 7B). TNF production decreased and IL-10 production increased in non-parenchymal cell culture supernatants from mice treated with Ly6C^-^ monocytes, and serum ALT level decreased (Fig 7C–7E). These data suggest that in the liver of *T*. *congolense*—infected mice, Ly6C^-^ monocytes promote the differentiation of Ly6C^+^ monocytes into macrophages and switch the cytokine milieu from hepatodestructive to hepatoprotective. This finding was confirmed by transferring GFP^+^ Ly6C^+^ monocytes or GFP^+^ Ly6C^+^ monocytes plus Ly6C^-^ monocytes isolated from infected WT mice in Nr4a1^-/-^ recipients that do not produce Ly6C^-^ monocytes (Fig 8A). In mice co-treated with Ly6C^-^ monocytes, the percentage of liver GFP^+^ cells that gained F4/80 and MHC-II expression and lowered their expression of CD11b, reflecting a differentiation towards macrophages, increased and coincided with reduced TNF and ALT level as well as with increased IL-10 level (Fig 8A–8D).

**Fig 9:**
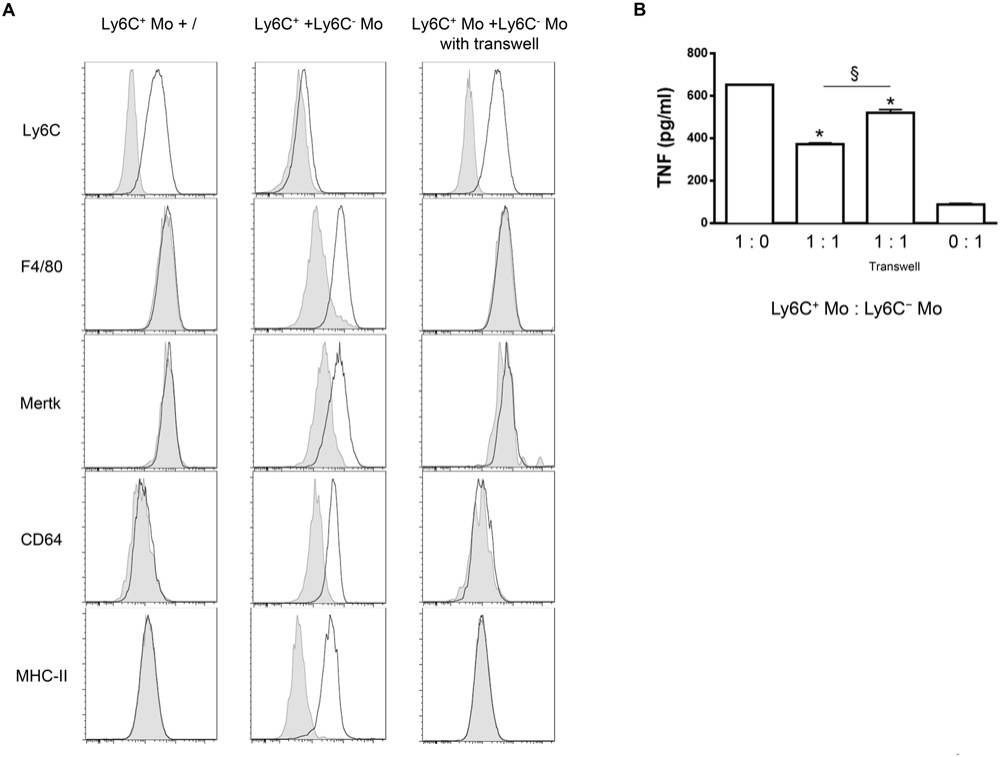
Differentiation of Ly6C^+^ monocytes into macrophages requires a physical contact with Ly6C^-^ monocytes. **(A)** Ly6C^+^ monocytes (Ly6C^+^ Mo) purified from *T*. *congolense*-infected CD45.2 WT mice at day 6 pi were cultured alone or with MHC-II^- to lo^ Ly6C- monocytes (Ly6C^-^ Mo) purified from infected CD45.2 LysM-GFP mice at day 21 pi (1: 1 ratio). Similar cultures were performed in transwell plates. Expression of Ly6C, F4/80, Mertk, CD64 or MHC-II (white line) was investigated 7 days later on GFP^-^ Ly6C^+^ monocyte-derived cells gated as in S5A Fig in S1 Text (grey line: isotype control). Results are representative of 1 out of 9 mice tested in 3 independent experiments. **(B)** TNF concentration in cell culture supernatants was measured. Data are shown as mean + SD of 1 representative out of three independent experiments. * p<0.05 compared to Ly6C^+^ monocytes cultured alone; § p<0.05 comparing populations linked by horizontal bar.

To address a mechanistic basis for how Ly6C^-^ monocytes could promote the differentiation of Ly6C^+^ monocytes into macrophages, Ly6C^+^ monocytes and GFP^+^ Ly6C^-^ monocytes purified from *T*. *congolense*-infected mice at day 6 and 21 pi, respectively, were co-cultured with and without a transwell plate. The Ly6C^+^ cell differentiation to macrophages was investigated after 7 days, gating out the GFP^+^ cells (Fig 9A). The GFP^-^ cell population exhibited reduced expression of Ly6C and increased expression of F4/80, MerTK, CD64 and MHC-II, reflecting macrophage differentiation of the Ly6C^+^ monocytes, yet only when the two monocyte populations were in contact. The capacity of Ly6C^-^ monocytes to inhibit the production of TNF by Ly6C^+^ monocytes was also partially ablated when the two cell populations were separated from each other (Fig 9B). Thus, a cell contact between the Ly6C^+^ and the Ly6C^-^ monocytes contributed to the differentiation of Ly6C^+^ cells into macrophages and to the inhibition of TNF production.

## Discussion

### Dynamics of liver myeloid cells in infected mice

As in other life-threatening liver inflammation models [26–28], liver cell necrosis is thought to be mainly mediated by TNF-producing Ly6C^+^ monocytes during experimental *T*. *congolense* infection in trypanotolerant C57BL/6 mice [7]. However, in trypanotolerant mice infected with this intravascular parasite, an IL-10-dependent, Foxp3^+^ Treg and CD11b^+^ myeloid cell-mediated immune response develops after the control of the first peak of parasitemia to regulate the inflammatory condition, hereby preserving liver integrity and preventing early death of the host. In this regard, our reported data suggest that myeloid cell-derived IL-10 only deactivates the pathogenic activity of M1-type Ly6C^+^ monocytes while Tregs affected both pathogenic T cells and myeloid cells [7, 11, 29]. Here, the heterogeneity and dynamic of liver myeloid cells in *T*. *congolense*-infected mice was further detailed. We now report that the mounted anti-inflammatory immune response was concomitant with the mobilization/accumulation in the liver of Ly6C^-^ monocytes (CX_3_CR1^hi^ CD11b^hi^ CD115^hi^ CD11a^hi^ F4/80^lo^ CD64^-^ CD11c^int^) and macrophages (CX3CR1^int^ CD11b^int^ F4/80^hi^ CD115^lo^ CD64^hi^ CD11c^-^) coupled with a decreased presence of Ly6C^+^ monocytes (CX_3_CR1^int^ CD11b^hi^ CD115^hi^ CD62L^hi^ F4/80^int^ CD64^lo^ CD11c^-^). The Ly6C^+^ monocytes and the macrophages mobilized in the liver of infected mice were prominently MHC-II^- to int^ and MHC-II^int to hi^, while the MHC-II^- to lo^ fraction of Ly6C^-^ monocytes mounted up. The switch from Ly6C^+^ monocytes to Ly6C^-^ monocytes and macrophages in the course of *T*. *congolense* infection reflected the change from a TNF, hepatodestructive to an IL-10, hepatoprotective milieu [11]. Accordingly, we show that Ly6C^+^ monocytes were the main producers of TNF in infected mice while Ly6C^-^ monocytes and macrophages contribute to IL-10 gene and protein expression. A similar sequential mobilization of TNF^+^ Ly6C^+^ and IL-10^+^ Ly6C^-^ myeloid cell subsets in the acute and chronic phase of various inflammation models has been highlighted [30–33].

### Fate and function of Ly6C^-^ monocytes in infected mice

It is commonly agreed that circulating Ly6C^+^ and Ly6C^-^ monocytes—depending on the pathogenic insult, their fate, timing of recruitment and location—exert tissue beneficial or detrimental functions. Hereby, Ly6C^+^ monocytes can infiltrate a variety of inflamed tissues, where they down-regulate their Ly6C expression and differentiate into M1- or M2-type macrophages that exert destructive or protective function in the initiation or resolving phase of inflammation, respectively [31–37]. Similarly, tissue-accumulating Ly6C^-^ monocytes can exert healing or damaging activity in different models of sterile and non-sterile inflammation [38–41]. While it was proposed that Ly6C^-^ monocytes can differentiate into macrophages or DCs in inflamed tissues [32, 38–43], it is now commonly agreed that even in steady state condition Ly6C^-^ monocytes are not bona fide monocytes but represent terminally differentiated "housekeeper" blood-resident macrophages crawling along the endothelium of the vessels [19, 38, 42, 44–47].

**Fig 10:**
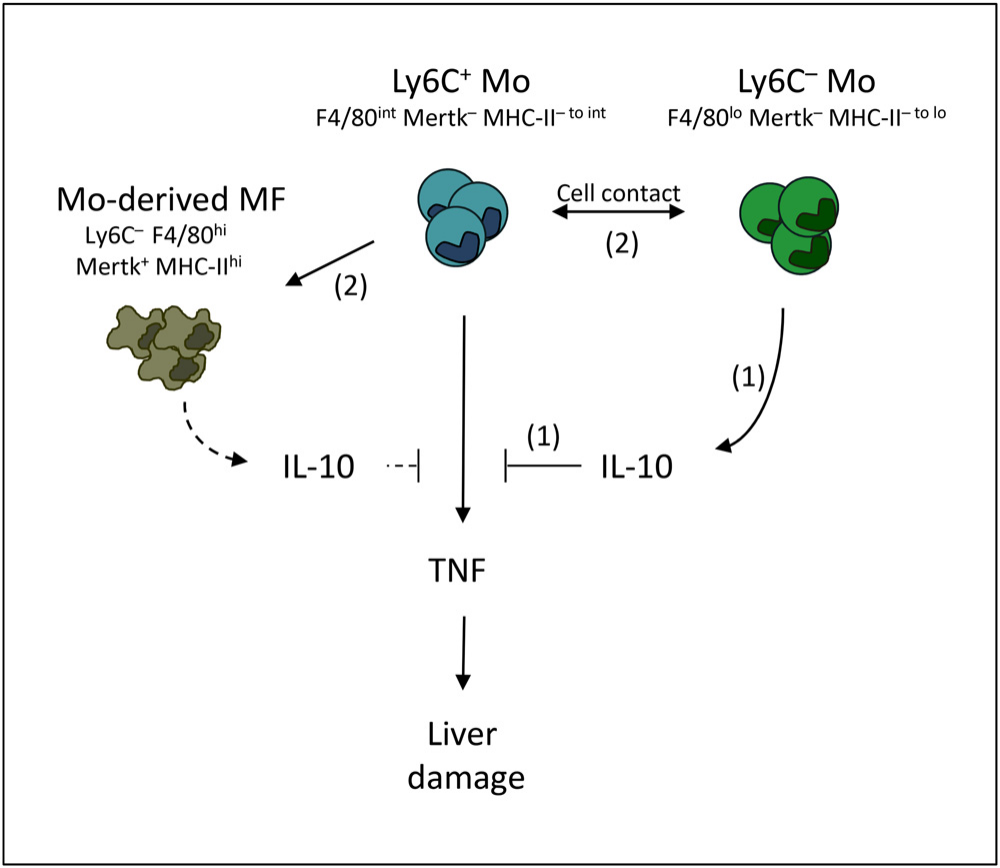
Overview of the interactions between Ly6C+ monocytes, Ly6C- monocytes and macrophages contributing to trypanotolerance. TNF-producing Ly6C^+^ monocytes contribute to liver injury in *T*. *congolense*-infected mice. Ly6C^-^ monocytes hamper the pathogenic activity of Ly6C^+^ monocytes through (1) IL-10 production and (2) cell contact that trigger the differentiation of Ly6C^+^ monocytes into restorative macrophages. Whether IL-10 possibly produced by monocyte-derived macrophages (dashed lines) contribute to hepatoprotective activity is not elucidated.

Using adoptive transfer experiments, we evidenced the differentiation of Ly6C^+^ monocytes mainly into Ly6C^-^ F4/80^hi^ Mertk^hi^ MHC-II^+^ CD11c^-^ Mar-1^-^ macrophages in the liver of *T*. *congolense* trypanotolerant mice (Fig 10). This differentiation pathway thus differed from the more commonly acknowledged differentiation of Ly6C^+^ monocytes into TIP DCs evidenced in trypanosusceptible mice or in bacterial infection [48, 49]. Similarly, in fibrotic liver, fibrogenic Ly6C^+^ monocytes rapidly differentiate into Ly6C^-^ "restorative" macrophages [33, 50]. Moreover, using BudU labelling, adoptive transfer experiments and CCR2^-/-^ mice, we confirmed that circulating CCR2-dependent Ly6C^+^ monocytes could also partially give rise to Ly6C^-^ monocytes in this inflammatory setting [19, 42, 44, 45]. The Ly6C^-^ monocytes that expanded during *T*. *congolense* infection did not seem to differentiate into macrophages or DCs as suggested by their lower *ex situ* expression of *Pu1*, *Cmaf* and *Mafb* genes and their low level of MHC-II molecule expression, as well as by maintenance of their F4/80^-^ MHC-II^- to lo^ phenotype after transfer in infected animals. However, we did not exclude that these cells were terminally differentiated macrophages [18, 19, 42, 44].

Very limited data exist about the functional role of Ly6C^-^ monocytes in the outcome of liver inflammation [51]. These cells do not seem to be mobilized in the liver during fibrosis progression [50], but a recent report suggests that the intrahepatic Ly6C^-^ monocyte population increases in a model of non-alcoholic fatty liver disease (NAFLD) provided mice receive a drug treatment that ameliorates the disease by decreasing inflammation [52]. We report here that that the liver Ly6C^-^ monocyte population expanded during *T*. *congolense* infection and exerted a hepatoprotective/restorative function. Indeed, the absence of Ly6C^-^ monocytes in infected Nr4a1^-/-^ mice resulted in higher TNF production and increased liver damage. In contrast, treatment of infected WT or Nr4a1^-/-^ mice with Ly6C^-^ monocytes triggered the differentiation of Ly6C^+^ monocytes into macrophages, limited TNF production, resulting in hepatoprotection. The limiting effect of Ly6C^-^ monocytes on TNF production by Ly6C^+^ monocytes was partially mediated by cell contact and by IL-10 (Fig 10). Indeed, the inhibition of TNF production was partially restored when the Ly6C^+^ monocytes and Ly6C^-^ monocytes were physically separated. Moreover, the treatment of infected mice with Ly6C^-^ monocytes from control IL-10^wt/wt^ LysM-cre^+/+^ mice reduced the production of TNF by Ly6C^+^ monocytes and the liver damage more efficiently than the treatment with Ly6C^-^ monocytes from IL-10^flox/flox^ LysM-cre^+/+^ mice lacking IL-10 gene expression in myeloid cells. Furthermore, the capacity of Ly6C^-^ monocytes to differentiate Ly6C^+^ monocytes into macrophages also required a cell contact. In this regard, PD-L1 or PD-1 surface expression by Ly6C^-^ monocytes was reported to trigger tolerogenic immune response [53, 54]. Moreover, soluble mediators like M-CSF, GM-CSF, IL-6, TGF-β, PGE2 and retinoic acid could contribute to the differentiation of Ly6C^+^ monocytes into macrophages and/or to inhibition of TNF production [32, 55–60].

The Ly6C^+^ monocytes, Ly6C^-^ monocytes and macrophages from *T*. *congolense*-infected mice exhibited a differential activation status. Ly6C^+^ monocytes expressed mainly M1-type genes, Ly6C^-^ monocytes expressed mainly IL-10 and macrophages expressed a mix of M1- and M2-type genes. Such a mixed expression of M1- and M2-type genes was previously associated with the restorative phenotype of anti-fibrotic liver macrophages [33]. The distinct gene profile of Ly6C^-^ monocytes and macrophages suggested that these cells exerted different functions in protecting the liver against *T*. *congolense*-induced injury. In this regard, macrophages expanding in the liver of *T*. *congolense*—infected mice expressed higher level than Ly6C^-^ monocytes of the M2-type gene selenoprotein P (*Sepp1*), that we documented to exert anti-oxidant hepatoprotective function during infection [10]. We could not ascertain directly that Ly6C^+^ monocyte-derived macrophages developed a restorative activation program in vivo because the low amount of adoptively transferred cells homing in the liver of infected mice did not consent their purification. Of note, it has become evident in the recent years that upon sterile and non-sterile inflammation, monocyte subsets originating from bone marrow and their derived macrophages complement the long-lived tissue-resident macrophage pool that originate from embryonic precursors [44, 61–64]. The current view is that in inflamed tissue, the resident macrophage pool restores homeostasis by local proliferation, by adopting a M2-type activation status and by secreting IL-10 [65–67]. Thus, besides monocyte-derived macrophages, tissue-resident macrophages are candidates to develop a restorative activation program during the late stage of *T*. *congolense* infection. However, no definitive reporter mice currently allow to discriminate the liver resident macrophages from the recruited monocyte-derived macrophages, hampering investigating their particular role, including M1/M2-type activation and proliferative status, in tissue pathogenicity.

In conclusion, we reveal that in the liver of *T*. *congolense*-infected mice Ly6C^-^ monocytes and macrophages were major sources of the anti-pathogenic cytokine IL-10. Ly6C^-^ monocytes exerted a hepatoprotective function through their IL-10 production and through cell-contact by limiting the TNF production by pathogenic Ly6C^+^ monocytes and by inducing the differentiation of the latter cells into restorative macrophages.

## Materials and Methods

### Ethic statement

Experiments, maintenance and care of mice complied with guidelines of the European Convention for the Protection of Vertebrate Animals used for Experimental and other Scientific Purposes (CETS n°123) and were approved by the Ethical Committee for Animal Experiments of the Vrije Universiteit Brussel, Brussels, Belgium (laboratory accreditation number 121 0220, project number 11-220-12).

### Mice, parasites, infections

CD45.2 C57BL/6 mice: CX_3_CR1-GFP^+/+^ mice were bred in house with CD45.2 WT mice purchased from Janvier, France to obtain CX_3_CR1-GFP^+/-^ mice. IL-10^flox/flox^ mice kindly donated by Dr. A. Roers (Department of Immunology, Technical University of Dresden, Germany) were crossed with IL-10^wt/wt^ LysM-cre^+/+^ mice (Jackson Laboratory, USA) to generate IL-10^flox/flox^ LysM- cre^+/+^ mice. LysM-GFP mice, kindly provided by Dr. T. Graf (Center for Genomic Regulation, Barcelona, Spain) and Nr4a1 (Nur77)^-/-^ mice were bred in house. CD45.1 WT C57BL/6 mice were bred in-house.

*T*. *congolense* variant antigen type 13 (Tc13, kindly provided by Dr. H. Tabel, University of Saskatchewan, Canada) stored at -80°C were injected in cyclophosphamide-treated WT C57BL/6 mice. At day 4 post infection, mice were bled, parasites were purified by diethyl-aminoethyl-cellulose chromatography and used to infect experimental female, age-matched (8–14 weeks) mice (2000 parasites/mouse, ip).

### Isolation of liver non-parenchymal immune cells and adoptive transfer of monocytes

Animals were euthanized (CO_2_) and livers were perfused through the portal vein with 10 ml of 120 U/ml collagenase type III (Worthington Biochemical Corporation) in HBSS (Gibco). Then, liver was disrupted mechanically and incubated in 10 ml of a 100 U/ml collagenase III solution in HBSS (25 min, 37°C). The resulting cell suspension was passed through a 70-μm nylon mesh filter and washed by adding 40 ml of HBSS supplemented with 10% FCS and 2 mM EDTA (HBSS/FCS/EDTA) followed by centrifugation (300 g, 8 min, 4°C). After erythrocyte lysis (0.83% NH_4_Cl in 0.01M Tris-HCl, pH 7.2), the pellet was resuspended in 10 ml of Lymphoprep (Lucron Bioproducts) and overlaid with 10 ml HBSS/FCS/EDTA. After centrifugation (430 g, 30 min, 20°C), the layer of low-density cells at the interface containing liver non-parenchymal cells was harvested, washed with HBSS containing 10% FCS (HBSS/FCS) and centrifuged (300 g, 10 min, 4°C). Cells were resuspended in HBSS/FCS, counted while excluding dead cells using trypan blue and diluted to working concentrations (5 x 10^6^/ml). Liver immune cells and buffers were kept on ice during isolation protocols and subsequent analysis. CD11b^+^ Ly6C^+^ Ly6G^+^ granulocytes were not retained within the non-parenchymal fractions due to their high-density characteristic.

At indicated time-points post infection, unfractionated CD11b^+^ liver immune cells were isolated using magnetic separation columns according to the manufacturer’s protocol (Miltenyi Biotec). BD FACSAria II (BD Biosciences) was then used to sort from CD11b^+^ cells, Ly6C^-^ monocytes from CX_3_CR1-GFP^+/-^ (CD11b^hi^ MHC-II^-^ CX_3_CR1^hi^), Ly6C^-^ monocytes from WT, IL-10^wt/wt^ LysM-cre^+/+^ or IL-10^flox/flox^ LysM-Cre^+/+^ mice (CD11b^hi^ CD115^+^ MHC-II^-^) or Ly6C^+^ monocytes from CX_3_CR1-GFP^+/-^, WT or LysM-GFP mice (CD11b^hi^ CD115^+^). Macrophages were sorted from CD11b^+^ cells of CX_3_CR1-GFP^+/-^ mice as CD115^+^ F4/80^hi^ Ly6C^-^ CX_3_CR1^int^ cells.

For adoptive transfer experiments, recipient mice were treated iv with Ly6C^-^ monocytes or Ly6C^+^ monocytes (8 x 10^5^ cells/mouse). Control mice were treated with HBSS. One day after the last transfer, liver immune cells were isolated and intracellular spontaneous production of TNF in Ly6C^+^ CD11b^+^ monocytes and Ly6C^-^ CD11b^+^ cells was analyzed by FACS. In addition, liver immune cells were cultured for 24 h and TNF concentration in supernatants was measured.

### Cell cultures and supernatants

At indicated time-points post infection, liver immune cells (2 x 10^6^/ml, 24-well plates) were cultured in RPMI 1640 (Gibco) supplemented with 10% FCS, 100 U/ml penicillin, 100 mg/ml streptomycin, 0.1 mM non-essential amino acids, 2 mM L-glutamine (all from Invitrogen Life Technologies). Alternatively, Ly6C^+^ and Ly6C^-^ monocytes were cultured at indicated ratio in 200 μl (96-well plates). Cell supernatants were collected 1 day later. When required, cultures were treated with 3 μg/ml of anti-IL10R antibody (clone 1B1.3a, kindly provided by Dr. O. Leo, ULB, Gosselies, Belgium) or control antibody (rat IgG1, BD Biosciences). For in vitro macrophage differentiation test, liver sorted Ly6C^+^ monocytes (2 x 10^5^) and GFP^+^ Ly6C^-^ monocytes (2 x 10^5^) were cultured without adding M-CSF in 200 μl medium. Cells and supernatants were harvested 7 days later. When required, Ly6C^+^ monocytes and GFP^+^ Ly6C^-^ monocytes were plated in the bottom and upper compartments of transwell plates (Costar, 0.4 μm), respectively.

### Quantification of cytokines

TNF and IL-10 was quantified in cell supernatants or in blood serum using specific sandwich ELISAs (R&D Systems), in accordance to the manufacturers’ protocols.

### Extracellular and intracellular FACS analysis

For extracellular stainings, liver immune cells (10^6^) were transferred in FACS tubes and stained for 20 min in ice-cold HBSS supplemented with 0.5% FCS using conventional protocols. Cells were pre-incubated with anti-FcγR antibody before adding commercial labeled surface marker antibodies (S1 Table in S1 Text). For intracellular stainings, cells (Ly6C^+^ CD11b^+^ monocytes, Ly6C^-^ CD11b^+^ cells) were cultured 4 h with brefeldin A (BD Biosciences), subsequently fixed and permeabilized following the manufacturer's instructions (BD Biosciences). Cells were then stained with PE-conjugated anti-TNF (clone MP6-XT22) or IgG1κ isotype control (R3-34) antibody and washed with ice-cold PBS. Cells were analyzed on a FACSCanto II (BD Biosciences). Delta-Median Fluorescence Intensity (dMFI) was calculated as difference between MFI of anti-TNF and isotype control antibody staining. Analyses were performed using FlowJo software.

### Quantitative real-time PCR

RNA extracted using the RNeasy plus mini kit (Qiagen) was reverse-transcribed with oligo(dT) and SuperScript II RT (Invitrogen), following the manufacturer's instructions. Quantitative real-time PCR was performed in an iCycle, with iQ SYBR Green Supermix (Bio-Rad). Primer sequences are listed in S2 Table in S1 Text. PCR cycles consisted of 1-minute denaturation at 94°C, 45-second annealing at 55°C, and 1-minute extension at 72°C. Gene expression was normalized using ribosomal S12 (Mrps12) protein as a housekeeping gene.

### Alanine aminotransferase (ALT) levels

ALT levels were measured in serum samples using a commercial kit (Boehringer).

### Statistical analysis

All comparisons were tested for statistical significance using the unpaired *t* test from GraphPad Prism 4.0 software.

## Supporting Information

S1 Text Contains **S1 Table** (Antibodies used for flow cytometry analysis) and **S2 Table** (Primers used for RT-PCR analyses) as supplement to Experimental procedures. The **S1 Text** contains also **S1 Fig** (Liver myeloid cells consist of 3 distinct populations in *T*. *congolense*-infected mice), **S2 Fig** (MHC-II expression on monocytes and macrophages from non-infected and infected mice), **S3 Fig** (BrdU and Ki67 labeling of monocytes and macrophages from infected mice), **S4 Fig** (Ly6C^-^ monocytes do not differentiate into macrophages in infected mice), **S5 Fig** (Accumulation of liver macrophages and Ly6C^-^ monocytes depends on CCR2 signalling in infected mice), **S6 Fig** (M2-type and M1-type gene expression levels in monocytes and macrophages from infected mice), **S7 Fig** (TNF production by non-parenchymal cells, Ly6C^+^ monocytes and macrophages from Nr4a1^-/-^ mice), **S8 Fig** (Percentage within non-parenchymal cells of monocytes and macrophages in infected Nr4a1^-/-^, WT and CCR2^-/-^ mice).
(PDF)
