# Supplementary material for: Ly6C- Monocytes Regulate Parasite-Induced Liver Inflammation by Inducing the Differentiation of Pathogenic Ly6C+ Monocytes into Macrophages
Source: PLoS Pathog. 2015 May 28;11(5):e1004873. doi: 10.1371/journal.ppat.1004873 (PMC4447383; doi:10.1371/journal.ppat.1004873)
Supplement: S1 Text — Contains S1 Table (Antibodies used for flow cytometry analysis) and S2 Table (Primers used for RT-PCR analyses) as supplement to Experimental procedures. The S1 Text contains also S1 Fig (Liver myeloid cells consist of 3 distinct populations in T. congolense-infected mice), S2 Fig (MHC-II expression on monocytes and macrophages from non-infected and infected mice), S3 Fig (BrdU and Ki67 labeling of monocytes and macrophages from infected mice), S4 Fig (Ly6C- monocytes do not differentiate into macrophages in infected mice), S5 Fig (Accumulation of liver macrophages and Ly6C- monocytes depends on CCR2 signalling in infected mice), S6 Fig (M2-type and M1-type gene expression levels in monocytes and macrophages from infected mice), S7 Fig (TNF production by non-parenchymal cells, Ly6C+ monocytes and macrophages from Nr4a1-/- mice), S8 Fig (Percentage within non-parenchymal cells of monocytes and macrophages in infected Nr4a1-/-, WT and CCR2-/- mice). (PDF) [file ppat.1004873.s001.pdf]

Ly6C⁻ monocytes regulate parasite-induced liver inflammation by inducing the differentiation of pathogenic Ly6C⁺ monocytes into macrophages

Yannick Morias et al.

Table S1: Antibodies used for extracellular flow cytometry analysis

Surface marker	Antibody clone	Label
CD16/32 (FcγR) [1]	2.4G2	none
CD11a [1]	2D7	PE
CD11b [1]	M1/70	PE-Cy7
CD11c [1]	HL3	PE
CD115 [1]	AF598	PE
CD49b [2]	Hma2	Biotin [5]
CD62L [1]	MEL-14	PE
CD64 [3]	X54-5/7.1	PE
F4/80 [1]	C1:A3-A	PE/FITC
FceR1 [2]	Mar-1	PE
Isotype IgM [1]	R4-22	PE
Isotype IgG1 [1]	R3-34	PE/FITC
Isotype IgG2a [1]	R35-95	PE/FITC
Isotype IgG2b [1]	A95-1	PE
Ly6C [1]	AL-21	APC
Ly6G [1]	1A8	APC-Cy7
Mertk [4]	108921	Biotin [5]
MHC II [3]	M5/114.15.2	Percp-Cy5.5
TNF [2]	MP6-XT22	PE
CD3 [1]	145-C2.11	APC-Cy7
B220 [1]	RA3-6B2	APC-Cy7
NK1.1 [2]	PK136	APC-Cy7

Antibodies manufactured by [1] BD Biosciences, [2] ebioscience, [3] Biolegend, [4] R&D Systems
[5] detected using APC-Cy7 streptavidin (BD Biosciences)

Table S2: Primers used for RT-PCR analyses [1]

Gene	Forward sequence	Reverse sequence
Arg1	5’-GCTGTCTTCCCAAGAGTTGGG-3’	5’-ATGGAAGAGACCTTCAGCTAC-3’
Ccl2	5’-CACTCACCTGCTGCTACTCATTCAC-3’	5’-GGATTCACAGAGAGGGAAAAATGG-3’
Cmaf	5’-GAGCCTTCTTCCCTTGACAG-3’	5’-ATTTAAAGTCCCCAAAGCA-3’
Ctss	5’-AGAAGGGCTGCGTCACTGAG-3’	5’-GAATGTACCTTGAACACGTAG-3’
Cxcl9	5’- TCAACAAAAGAGCTGCCAAA-3’	5’- GCAGAGGCCAGAAGAGAGAA-3’
Cxcl10	5’-AGATCATTGCCACGATGAAA-3’	5’-CACTGGGTAAAGGGGAGTGA-3’
Folr2	5’-GGAGCTACACAAGGCTGAC-3’	5’-TGTGACAGGGTGCTGTGTTT-3’
F13a	5’-CCAGGAATTAAGCAAGACATC-3’	5’-TGCCCTTACTTTCTAGTTCTC-3’
Il10	5’-ACTCAATACACACTGCAGGTG-3’	5’-GGACTTTAAGGGTTACTTGG-3’
Mafb	5’-TCGTCCTTCCCACTTCAGTT-3’	5’-ACGCAAAGCCTGTCTTGTTT-3’
Mgl2	5’- CGGCTGGAGAATTCTCACCT-3’	5’- TAATGCCTCTGGCAGACATC-3’
Ngfb	5’-CTTCTCATCTGTTGTCAACGC-3’	5’-CATAGCGTAATGTCCATGTTGTTCT-3’
Nos2	5’-TGGAGCCAAGGCCAAACACAG-3’	5’-TCCACCAGGAGATGTTGAAC-3’
Nr4a1	5'-AGCTTGGGTGTTGATGTTCC-3'	5'-TAAAGGCACATGGGTGACAG-3'
Pu.1	5’-GATTCGCCTGTACCAGTTCC-3’	5’-TTCTTCACCTCGCCTGTCTT-3’
Relb	5’-AATCGAGAGCAAACGAAGGA-3’	5’-CCCACAAGGCTAGCAGTACC-3’
Sepp1	5’-TTCTGCAGGCATCCAGATTG-3’	5’-CACAAGACGGCCACATCTGT-3’
S12	5’-GGAAGGCATAGCTGCTGGAGGTGT-3’	5’-CCTCGATGACATCCTTGGCCTGAG-3’
Tnf	5’-CCTTCACAGAGCAATGACTC-3’	5’-GTCTACTCCCAGGTTCTCTTC-3’

[1] purchased at Sigma, designed using the Primer-Blast software (NCBI)

Fig S1: Liver myeloid cells from *T. congolense*-infected mice consist of 3 distinct populations

(A) Gating strategy: $CD11b^+$ $(Ly6G/CD49b/CD3/B220/NK1.1)^-$ cells in liver non-parenchymal cell fraction from $CX_3CR1\text{-}GFP^{+/-}$ mice at d21 pi were analyzed for expression of Ly6C and CX_3CR1/GFP. **(B)** Expression of indicated surface markers (■) was investigated on $Ly6C^+$ monocytes ($Ly6C^+$ Mo), $Ly6C^-$ monocytes ($Ly6C^-$ Mo) and macrophages (MF) gated as in (A)(□: isotype control). The nature of the myeloid cell subsets was confirmed by addressing their forward (FSC) and side scatter (SSC) and their gene expression of dedicated transcription factors. **(C)** FSC/SSC profiles of $Ly6C^+$ monocytes (□), $Ly6C^-$ monocytes (■) and macrophages (■). Results are representative of 1 out of 18 mice tested in 5 independent experiments. **(D)** *Pu1*, *Nr4a1*, *Mafb* and *Cmaf* gene expression in FACS-sorted $Ly6C^+$ monocytes, $Ly6C^-$ monocytes and macrophages normalized against *S12* gene expression and expressed relatively to gene expression in non-fractionated $CD11b^+$ liver cells. *Pu.1* that directs $Ly6C^+$ monocyte development and macrophage differentiation [1, 2] was expressed more in $Ly6C^+$ monocytes and in macrophages than in $Ly6C^-$ monocytes; *Nr4a1* (*Nur77*) expression that regulates the differentiation and survival of $Ly6C^-$ monocytes [3] was higher in the $Ly6C^-$ monocytes than in the $Ly6C^+$ monocytes and the macrophages; and *Mafb* and *Cmaf* that promote macrophage terminal differentiation linked to a cell cycle exit [4-6] were higher expressed in macrophages than in the $Ly6C^+$ and $Ly6C^-$ monocyte populations. Data are shown as mean + SD of 3 individual mice from one representative out of three independent experiments. # $p<0.05$ compared to non-fractionated $CD11b^+$ myeloid cells; * $p<0.05$ comparing populations linked by horizontal bar.

Fig S2: $Ly6C^+$ monocytes, $Ly6C^-$ monocytes and macrophages express different levels of MHC-II in *T. congolense*-infected mice

Expression of MHC-II molecule and SSC was investigated on $Ly6C^+$ monocytes ($Ly6C^+$ Mo), $Ly6C^-$ monocytes ($Ly6C^-$ Mo) and macrophages (MF) gated as in S1A Fig in the blood and the liver of non-infected and infected CX_3CR1-$GFP^{+/-}$ mice at day 7, 14 and 21 pi. Results are representative of 1 out of 12 mice tested in 4 independent experiments.

Fig S3: $Ly6C^+$ monocytes, $Ly6C^-$ monocytes and macrophages exhibit distinct turn over in *T. congolense*-infected mice

CX_3CR1-$GFP^{+/-}$ mice were treated ip with BrdU at day 11 pi. BrdU was then constantly administered via drinking water. After 6, 24, 48, 96 and 168 hours, **(A)** percentages within blood or liver non-parenchymal cells of $BrdU^+$ $Ly6C^+$ monocytes ($Ly6C^+$ Mo), $Ly6C^-$ monocytes ($Ly6C^-$ Mo) and macrophages (MF) gated as in S1A Fig were determined using anti-BrdU antibody. Data are shown as mean ± SD of 3 individual mice from one representative out of two independent experiments. * $p<0.05$ as compared to blood $Ly6C^+$ monocytes. # $p<0.05$ as compared to blood or liver $Ly6C^+$ monocytes. § $p<0.05$ as compared to liver $Ly6C^+$ monocytes. **(B)** Expression of Ki67, measured using anti-Ki67 antibody in $Ly6C^+$ monocytes, $Ly6C^-$ monocytes and macrophages from BrdU-treated mice. Control = Ki67 isotype control antibody staining versus BrdU staining in mice not treated with BrdU; representative of 1 out of 9 mice tested in three independent experiments)

Fig S4: $Ly6C^-$ monocytes do not differentiate into macrophages in *T. congolense*-infected mice

Liver GFP^+ $Ly6C^-$ monocytes ($Ly6C^-$ Mo) purified from CD45.2 CX_3CR1-$GFP^{+/-}$ mice at day 21 pi were transferred in CD45.1 WT mice at day 21 pi. After 96 h, liver $CD45.2^+$ cells in recipient mice were analyzed for Ly6C and CX_3CR1/GFP expression. MHC-II and F4/80 expression was then investigated in $Ly6C^-$ CX3CR1/GFP^+ cells. FACS profiles are

representative of 1 out of 9 mice tested in three independent experiments. Percentages of cells in indicated gates are as shown as mean ± SD of 3 individual mice of one representative out of three independent experiments.

Fig S5: Accumulation of liver macrophages and Ly6C$^-$ monocytes depends on CCR2 signalling in *T. congolense*-infected mice

(A) Gating strategy for liver Ly6C$^+$ monocytes (Ly6C$^+$ Mo), Ly6C$^-$ monocytes (Ly6C$^-$ Mo) and macrophages (MF) in WT and CCR2$^{-/-}$ mice at day d21 pi based on differential expression of CD11b, CD115, Ly6C, MHC-II and F4/80. **(B)**. Validation of the gating strategy in liver non-parenchymal cells from CD45.2 CX_3CR1-GFP$^{+/-}$ mice at d21 pi: **(1)** Monocytes and macrophages were selected based on co-expression of CD11b and CD115. **(2)** Ly6C and MHC-II expression on CD11b$^+$ CD115$^+$ cells was then used to discriminate Ly6C$^+$ monocytes from Ly6C$^-$ monocyte and macrophage populations. Since Ly6C$^-$ monocytes and macrophages expanding during infection were MHC-II$^{-\text{ to lo}}$ and MHC-II$^{\text{int to hi}}$, respectively (Fig 1, S2 Fig), MHC-II expression allowed distinguishing these 2 populations. **(3)** Ly6C and CX_3CR1/GFP expression in populations gated as in **(A)** confirmed the use of CD11b, CD115, Ly6C, MHC-II and F4/80 co-staining as alternative selection procedure for Ly6C$^+$ monocytes (Ly6C$^+$ Mo), Ly6C$^-$ monocytes (Ly6C$^-$ Mo) and macrophages (MF) in mice that do not express GFP reporter gene (S1A Fig). **(C,D)** Numbers in WT and CCR2$^{-/-}$ mice at day 7 and 21 pi of Ly6C$^+$ monocytes (Ly6C$^+$ Mo) and Ly6C$^-$ monocytes (Ly6C$^-$ Mo) in the blood and of Ly6C$^+$ monocytes, Ly6C$^-$ monocytes and macrophages (MF) within liver non-parenchymal cell population gated as in panel **(A)**. In the blood and the liver of both WT and CCR2$^{-/-}$ mice, Ly6C$^+$: Ly6C$^-$ monocyte ratio is in favor of Ly6C$^+$ monocytes or Ly6C- monocytes during the tissue-destructive (day 7 pi) or tissue-restorative (day 21 pi) stage of infection, respectively. Data are shown as mean + SD of 3 individual mice from one representative out of three independent experiments. * $p< 0.05$ comparing populations WT and CCR2$^{-/-}$ mice.

Fig S6: Liver myeloid cell subsets from *T. congolense*-infected mice exhibit distinct gene expression profile

Gene expression of indicated genes in liver Ly6C^{+} monocytes (Ly6C^{+} Mo), Ly6C^{-} monocytes (Ly6C^{-} Mo) and macrophages (MF) purified from CX_3CR1-GFP$^{+/-}$ mice at day 21 pi was normalized against *S12* gene expression and expressed relatively to gene expression in non-fractionated CD11b^{+} liver cells (**(A)** IL-10-dependent M2-type genes; (**B)** IL-10-inhibited M1-type genes). Data are shown as mean + SD of 3 individual mice from one representative out of three independent experiments. # $p<0.05$ compared to non-fractionated CD11b^{+} myeloid cells; § $p<0.05$ comparing populations linked by horizontal bar.

Fig S7: Absence of Nr4a1 expression does not affect TNF production by liver Ly6C^{+} monocytes and macrophages

Liver non parenchymal cell were isolated from non-infected WT and Nr4a1$^{-/-}$ mice. **(A)** Spontaneous and LPS-induced TNF concentration in non-parenchymal cell supernatants was measured (n.d., non-detected). **(B)** Percentage of spontaneous TNF producing Ly6C^{+} monocytes (Ly6C^{+} Mo) and macrophages (MF) gated as in S5A Fig within liver non-parenchymal cells (NPC) was determined by intracellular FACS staining. **(C)** Spontaneous TNF level in Ly6C^{+} monocytes and macrophages was determined as mean fluorescence intensity difference (dMFI) between anti-TNF and isotype control antibodies. Data are shown as mean + SD of 1 representative out of three independent experiments.

Fig S8: Percentages of liver Ly6C^{+} monocytes, Ly6C^{-} monocytes and macrophages in *T. congolense*-infected Nr4a1$^{-/-}$, WT and CCR2$^{-/-}$ mice

Percentages of liver Ly6C$^+$ monocytes (Ly6C$^+$ Mo), Ly6C$^-$ monocytes (Ly6C$^-$ Mo) and macrophages (MF) within liver non-parenchymal cell (NPC) population were determined **(A)** in WT and Nr4a1$^{-/-}$ mice at day 21 pi. For comparison, cell percentages are shown in **(B)** non-infected (n.i) and infected CX_3CR1-GFP$^{+/-}$ mice at day 7, 14 and 21 pi, and **(C)** in WT and CCR2$^{-/-}$ mice at day 7 and 21 post infection. Cell populations were gated as in S5A Fig **(A, C)** or S1A Fig (**B)**. Data are shown as mean + SD of 3 individual mice from one representative out of four independent experiments. * $p< 0.05$ compared to WT infected mice; § $p<0.05$ compared to non-infected mice.

References

1. DeKoter RP, Walsh JC, Singh H. PU.1 regulates both cytokine-dependent proliferation and differentiation of granulocyte/macrophage progenitors. EMBO J. 1998;17(15):4456-68.
2. Dahl R, Walsh JC, Lancki D, Laslo P, Iyer SR, Singh H, et al. Regulation of macrophage and neutrophil cell fates by the PU.1:C/EBPalpha ratio and granulocyte colony-stimulating factor. Nat Immunol. 2003;4(10):1029-36.
3. Hanna RN, Carlin LM, Hubbeling HG, Nackiewicz D, Green AM, Punt JA, et al. The transcription factor NR4A1 (Nur77) controls bone marrow differentiation and the survival of Ly6C- monocytes. Nat Immunol. 2011;12(8):778-85.
4. Aziz A, Soucie E, Sarrazin S, Sieweke MH. MafB/c-Maf deficiency enables self-renewal of differentiated functional macrophages. Science. 2009;326(5954):867-71.
5. Hegde SP, Zhao J, Ashmun RA, Shapiro LH. c-Maf induces monocytic differentiation and apoptosis in bipotent myeloid progenitors. Blood. 1999;94(5):1578-89.
6. Kelly LM, Englmeier U, Lafon I, Sieweke MH, Graf T. MafB is an inducer of monocytic differentiation. EMBO J. 2000;19(9):1987-97.

Fig S1

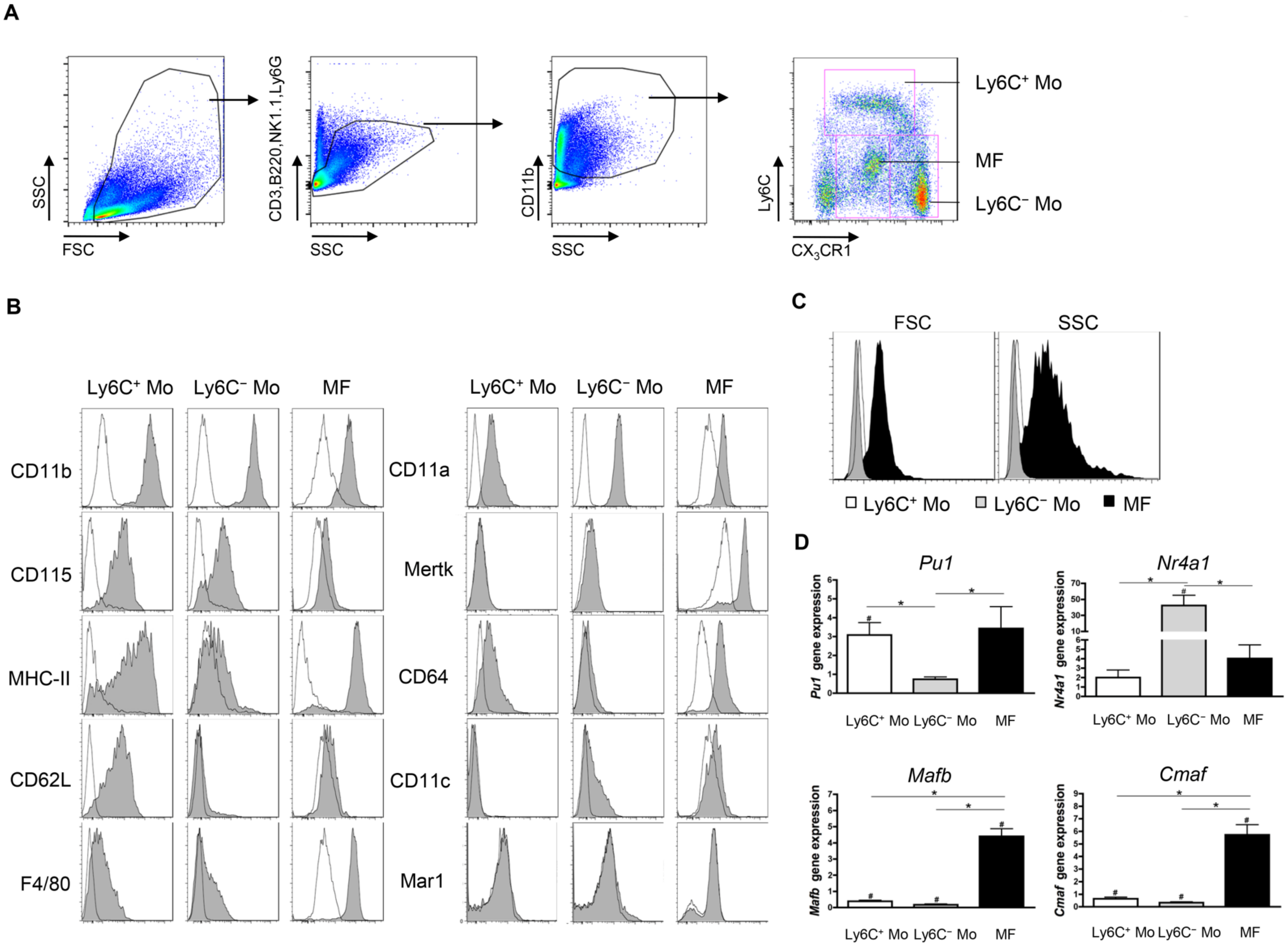
A
SSC
FSC
CD3,B220,NK1.1,Ly6G
SSC
CD11b
SSC
Ly6C
CX3CR1
Ly6C⁺ Mo
MF
Ly6C⁻ Mo
B
Ly6C⁺ Mo
Ly6C⁻ Mo
MF
CD11b
CD115
MHC-II
CD62L
F4/80
CD11a
Mertk
CD64
CD11c
Mar1
C
FSC
SSC
Ly6C⁺ Mo
Ly6C⁻ Mo
MF
D
Pu1
Pu1 gene expression
Nr4a1
Nr4a1 gene expression
Mafb
Mafb gene expression
Cmaf
Cmaf gene expression

Fig S2

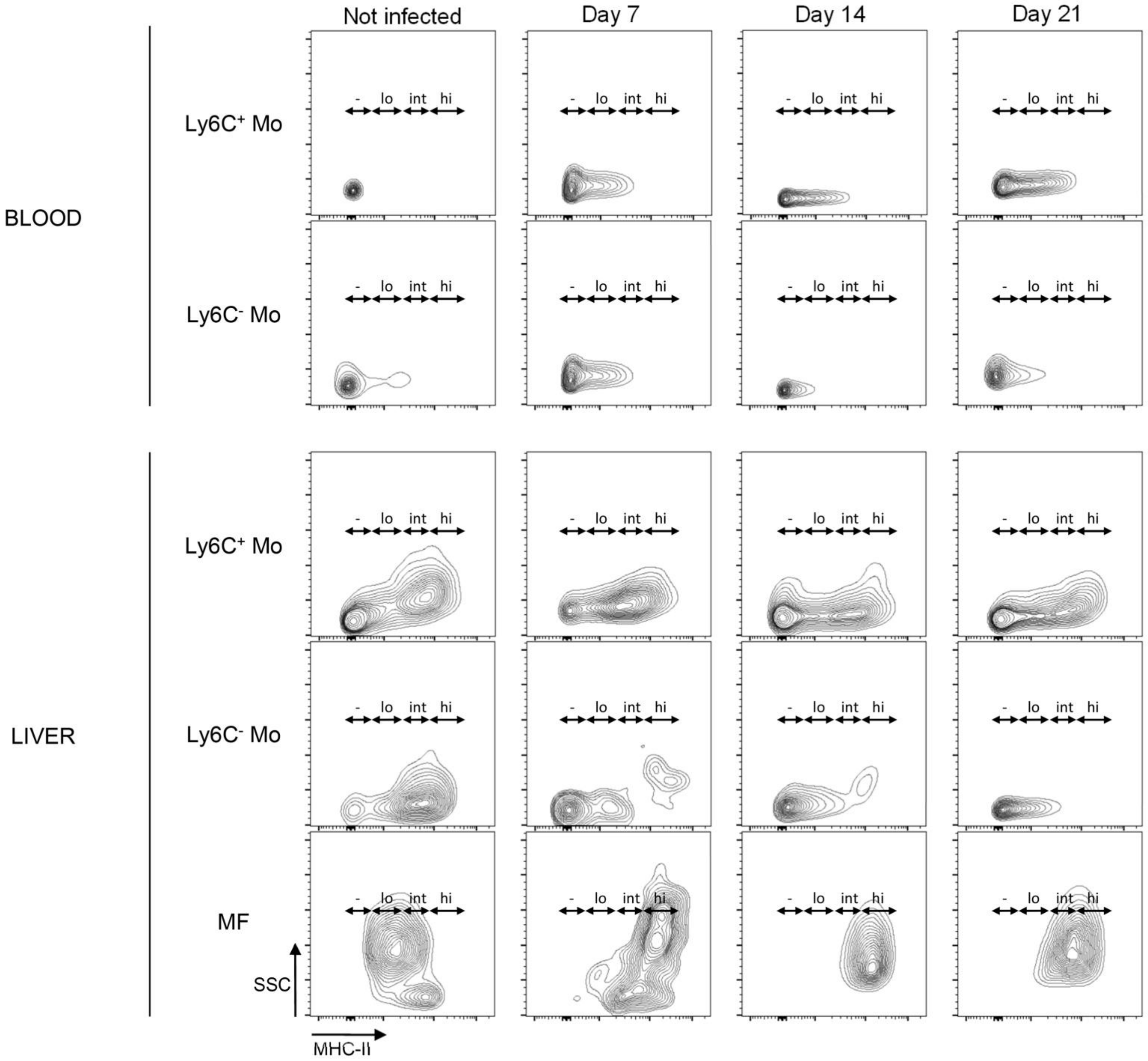
Not infected
Day 7
Day 14
Day 21
BLOOD
Ly6C+ Mo
Ly6C- Mo
LIVER
Ly6C+ Mo
Ly6C- Mo
MF
- lo int hi
SSC
MHC-II

Fig S3

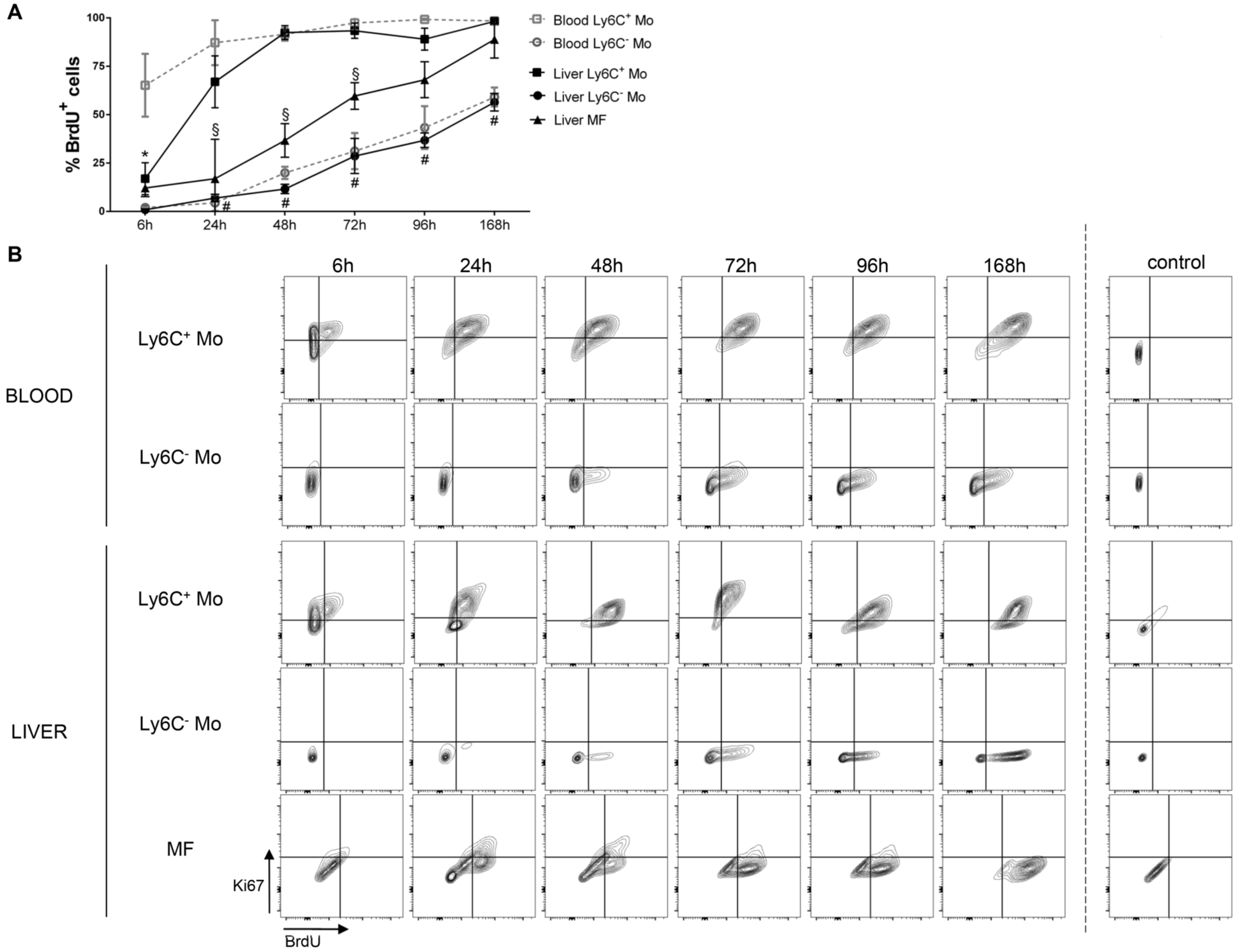
A
% BrdU+ cells
100
75
50
25
0
6h
24h
48h
72h
96h
168h
Blood Ly6C+ Mo
Blood Ly6C- Mo
Liver Ly6C+ Mo
Liver Ly6C- Mo
Liver MF
B
6h
24h
48h
72h
96h
168h
control
BLOOD
Ly6C+ Mo
Ly6C- Mo
LIVER
Ly6C+ Mo
Ly6C- Mo
MF
Ki67
BrdU

Fig S4

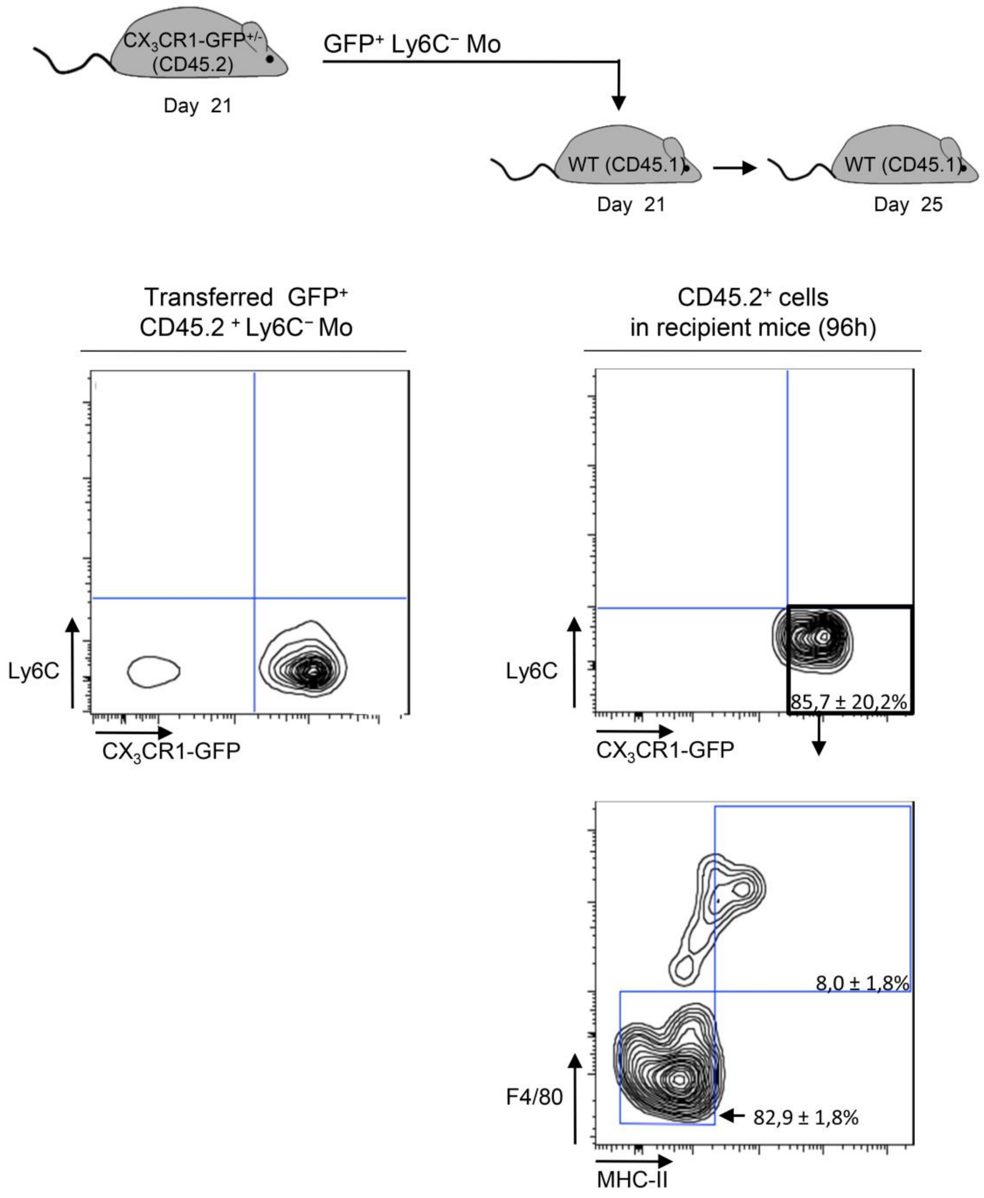
CX3CR1-GFP+/-
(CD45.2)
Day 21
GFP+ Ly6C− Mo
WT (CD45.1)
Day 21
WT (CD45.1)
Day 25
Transferred GFP+
CD45.2 + Ly6C− Mo
Ly6C
CX3CR1-GFP
CD45.2+ cells
in recipient mice (96h)
Ly6C
85,7 ± 20,2%
CX3CR1-GFP
8,0 ± 1,8%
F4/80
82,9 ± 1,8%
MHC-II

Fig S5

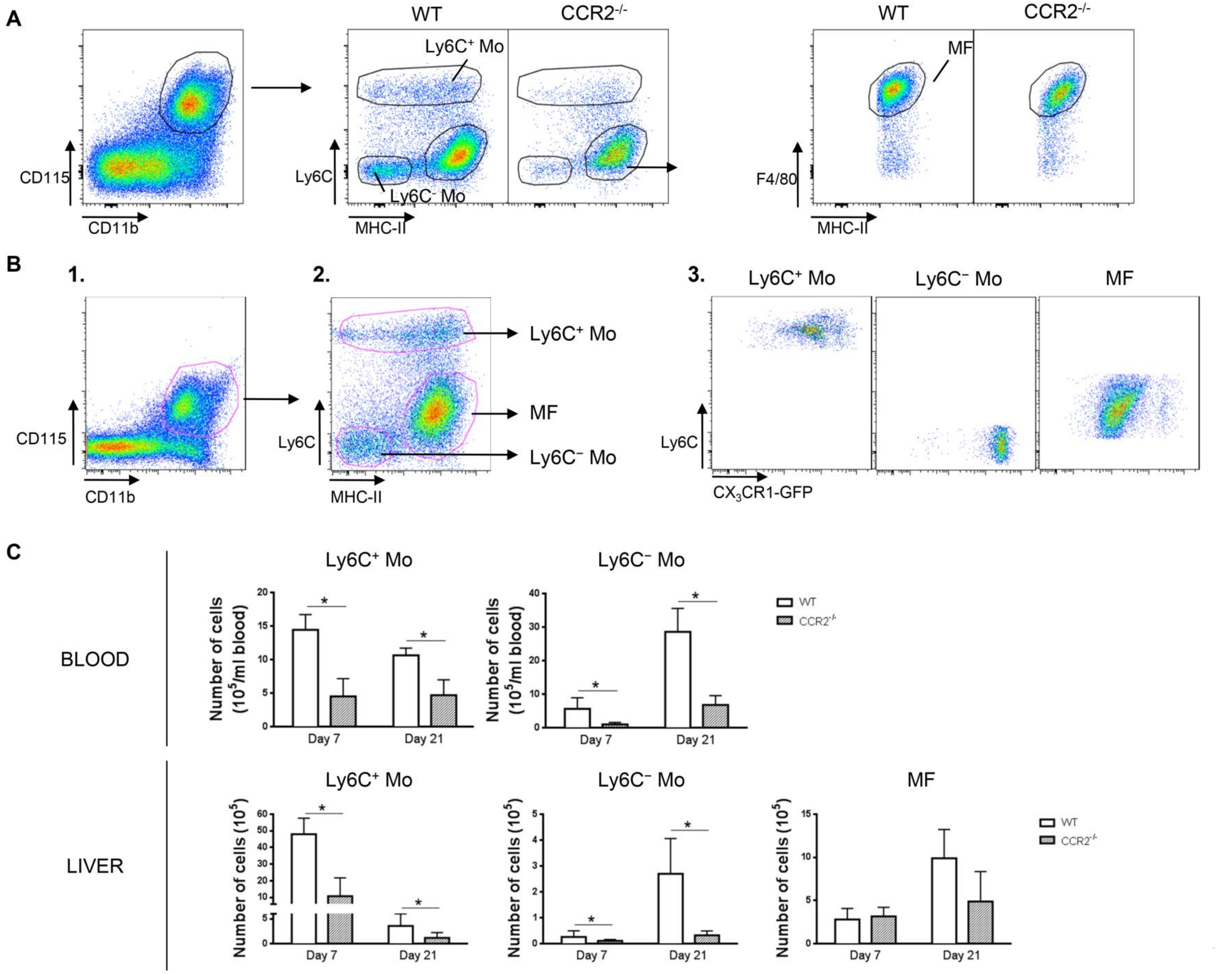
A
WT
CCR2-/-
Ly6C+ Mo
Ly6C- Mo
MF
CD115
CD11b
Ly6C
MHC-II
F4/80
B
1.
2.
3.
Ly6C+ Mo
MF
Ly6C− Mo
CX3CR1-GFP
C
BLOOD
LIVER
Number of cells (10^5/ml blood)
Number of cells (10^5)
Day 7
Day 21
WT
CCR2-/-

Fig S6

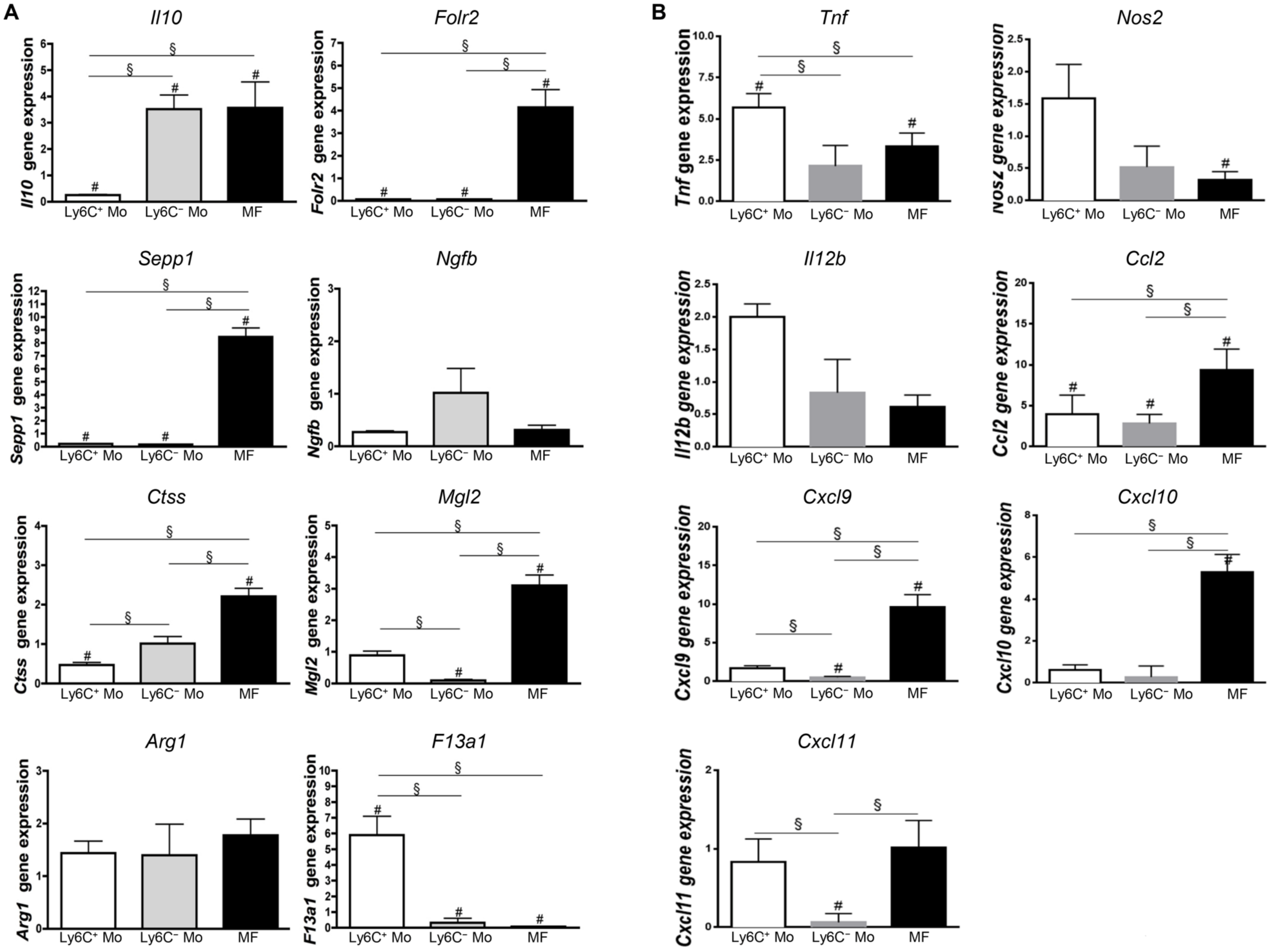
A
Il10
Il10 gene expression
Ly6C⁺ Mo
Ly6C⁻ Mo
MF
Folr2
Folr2 gene expression
Sepp1
Sepp1 gene expression
Ngfb
Ngfb gene expression
Ctss
Ctss gene expression
Mgl2
Mgl2 gene expression
Arg1
Arg1 gene expression
F13a1
F13a1 gene expression
B
Tnf
Tnf gene expression
Nos2
nos2 gene expression
Il12b
Il12b gene expression
Ccl2
Ccl2 gene expression
Cxcl9
Cxcl9 gene expression
Cxcl10
Cxcl10 gene expression
Cxcl11
Cxcl11 gene expression

Fig S7

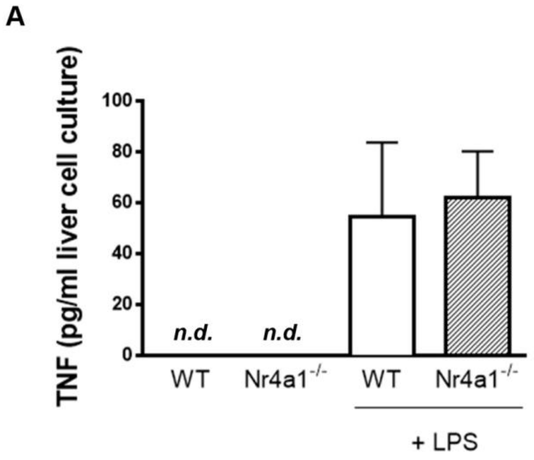
A
TNF (pg/ml liver cell culture)
100
80
60
40
20
0
n.d.
n.d.
WT
Nr4a1-/-
WT
Nr4a1-/-
+ LPS

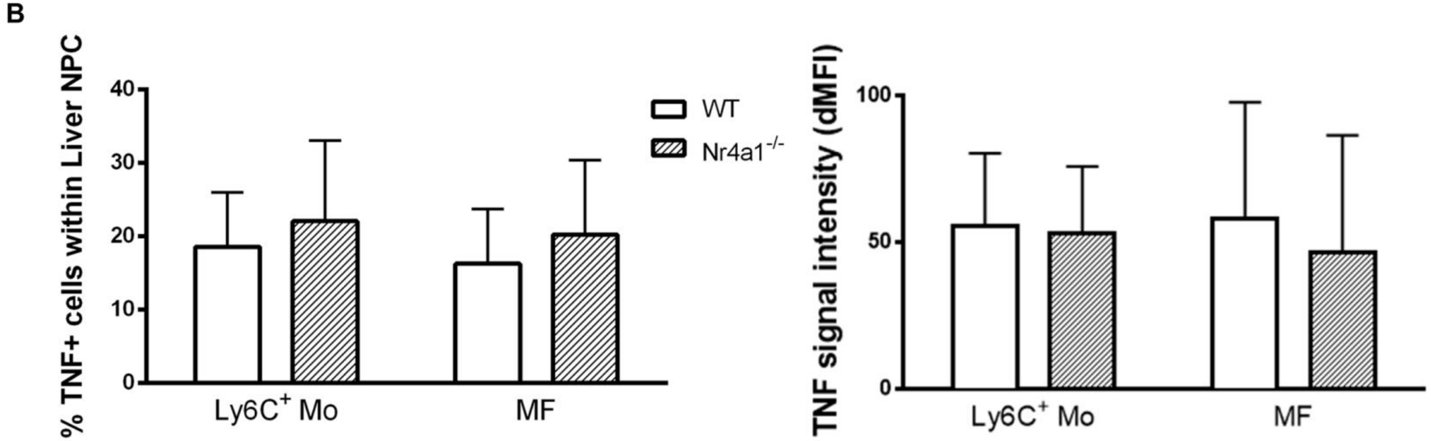
B
% TNF+ cells within Liver NPC
40
30
20
10
0
Ly6C+ Mo
MF
WT
Nr4a1-/-
TNF signal intensity (dMFI)
100
50
0
Ly6C+ Mo
MF

Fig S8

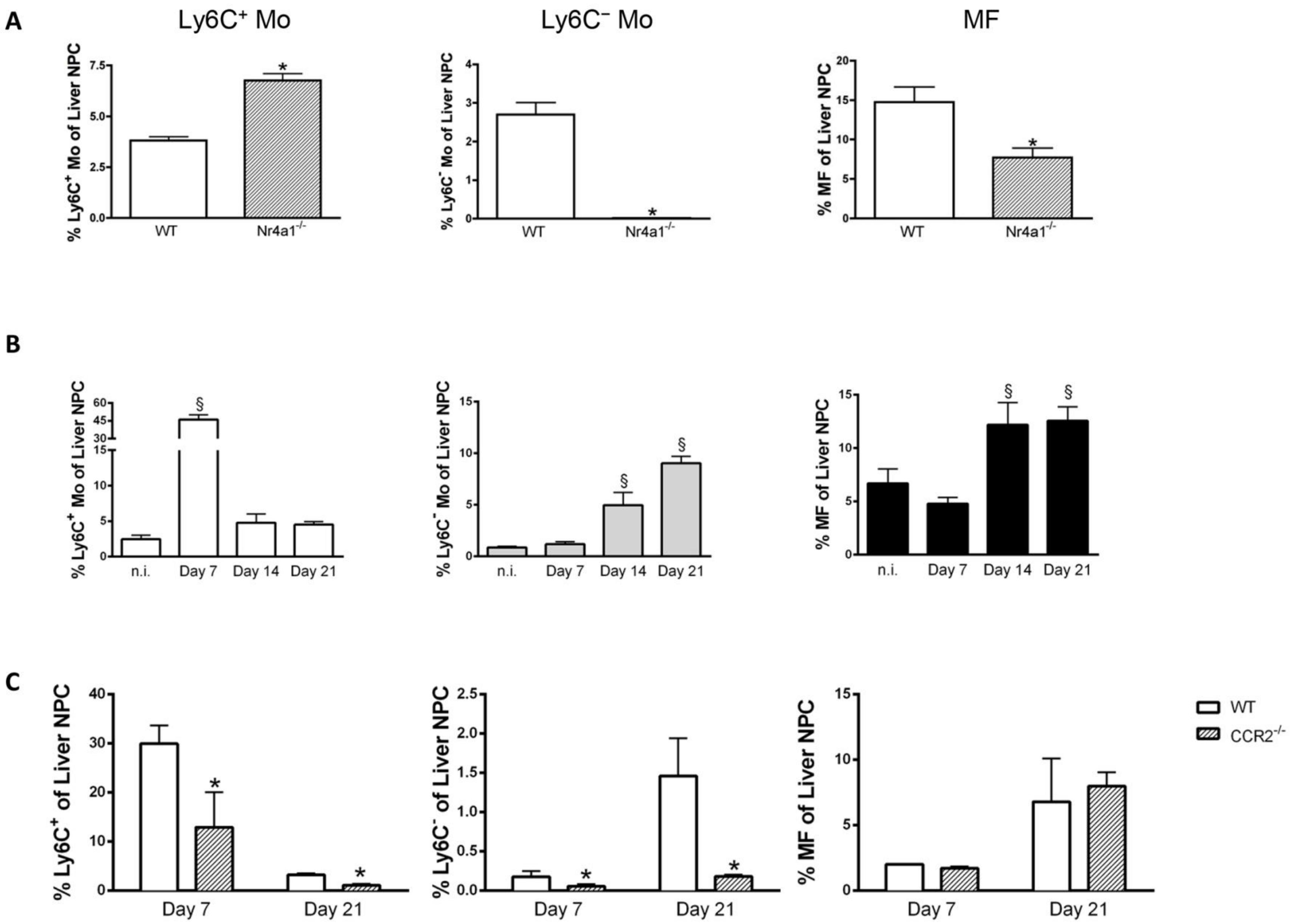
A
Ly6C⁺ Mo
% Ly6C⁺ Mo of Liver NPC
WT
Nr4a1-/-
Ly6C⁻ Mo
% Ly6C⁻ Mo of Liver NPC
MF
% MF of Liver NPC
B
n.i.
Day 7
Day 14
Day 21
C
% Ly6C⁺ of Liver NPC
% Ly6C⁻ of Liver NPC
WT
CCR2-/-
